# Lifetime Medical Costs of Obesity: Prevention No Cure for Increasing Health Expenditure

**DOI:** 10.1371/journal.pmed.0050029

**Published:** 2008-02-05

**Authors:** Pieter H. M van Baal, Johan J Polder, G. Ardine de Wit, Rudolf T Hoogenveen, Talitha L Feenstra, Hendriek C Boshuizen, Peter M Engelfriet, Werner B. F Brouwer

**Affiliations:** 1 National Institute for Public Health and the Environment (RIVM), Centre for Prevention and Health Services Research, Bilthoven, The Netherlands; 2 National Institute for Public Health and the Environment, Centre for Public Health Forecasting, Bilthoven, The Netherlands; 3 Tilburg University, Department Tranzo, Tilburg, The Netherlands; 4 Erasmus University, Medical Center, Rotterdam, The Netherlands; London School of Hygiene & Tropical Medicine, United Kingdom

## Abstract

**Background:**

Obesity is a major cause of morbidity and mortality and is associated with high medical expenditures. It has been suggested that obesity prevention could result in cost savings. The objective of this study was to estimate the annual and lifetime medical costs attributable to obesity, to compare those to similar costs attributable to smoking, and to discuss the implications for prevention.

**Methods and Findings:**

With a simulation model, lifetime health-care costs were estimated for a cohort of obese people aged 20 y at baseline. To assess the impact of obesity, comparisons were made with similar cohorts of smokers and “healthy-living” persons (defined as nonsmokers with a body mass index between 18.5 and 25). Except for relative risk values, all input parameters of the simulation model were based on data from The Netherlands. In sensitivity analyses the effects of epidemiologic parameters and cost definitions were assessed. Until age 56 y, annual health expenditure was highest for obese people. At older ages, smokers incurred higher costs. Because of differences in life expectancy, however, lifetime health expenditure was highest among healthy-living people and lowest for smokers. Obese individuals held an intermediate position. Alternative values of epidemiologic parameters and cost definitions did not alter these conclusions.

**Conclusions:**

Although effective obesity prevention leads to a decrease in costs of obesity-related diseases, this decrease is offset by cost increases due to diseases unrelated to obesity in life-years gained. Obesity prevention may be an important and cost-effective way of improving public health, but it is not a cure for increasing health expenditures.

## Abstract 2

Using a simulation model, Pieter van Baal and colleagues conclude that obesity prevention leads to a decrease in costs of obesity-related diseases, but this is offset by cost increases due to diseases unrelated to obesity in life-years gained.

## Editors' Summary

**Background.:**

Since the mid 1970s, the proportion of people who are obese (people who have an unhealthy amount of body fat) has increased sharply in many countries. One-third of all US adults, for example, are now classified as obese, and recent forecasts suggest that by 2025 half of US adults will be obese. A person is overweight if their body mass index (BMI, calculated by dividing their weight in kilograms by their height in meters squared) is between 25 and 30, and obese if BMI is greater than 30. Compared to people with a healthy weight (a BMI between 18.5 and 25), overweight and obese individuals have an increased risk of developing many diseases, such as diabetes, coronary heart disease and stroke, and tend to die younger. People become unhealthily fat by consuming food and drink that contains more energy than they need for their daily activities. In these circumstances, the body converts the excess energy into fat for use at a later date. Obesity can be prevented, therefore, by having a healthy diet and exercising regularly.

**Why Was This Study Done?:**

Because obesity causes so much illness and premature death, many governments have public-health policies that aim to prevent obesity. Clearly, the improvement in health associated with the prevention of obesity is a worthwhile goal in itself but the prevention of obesity might also reduce national spending on medical care. It would do this, the argument goes, by reducing the amount of money spent on treating the diseases for which obesity is a risk factor. However, some experts have suggested that these short-term savings might be offset by spending on treating the diseases that would occur during the extra lifespan experienced by non-obese individuals. In this study, therefore, the researchers have used a computer model to calculate yearly and lifetime medical costs associated with obesity in The Netherlands.

**What Did the Researchers Do and Find?:**

The researchers used their model to estimate the number of surviving individuals and the occurrence of various diseases for three hypothetical groups of men and women, examining data from the age of 20 until the time when the model predicted that everyone had died. The “obese” group consisted of never-smoking people with a BMI of more than 30; the “healthy-living” group consisted of never-smoking people with a healthy weight; the “smoking” group consisted of lifetime smokers with a healthy weight. Data from the Netherlands on the costs of illness were fed into the model to calculate the yearly and lifetime health-care costs of all three groups. The model predicted that until the age of 56, yearly health costs were highest for obese people and lowest for healthy-living people. At older ages, the highest yearly costs were incurred by the smoking group. However, because of differences in life expectancy (life expectancy at age 20 was 5 years less for the obese group, and 8 years less for the smoking group, compared to the healthy-living group), total lifetime health spending was greatest for the healthy-living people, lowest for the smokers, and intermediate for the obese people.

**What Do These Findings Mean?:**

As with all mathematical models such as this, the accuracy of these findings depend on how well the model reflects real life and the data fed into it. In this case, the model does not take into account varying degrees of obesity, which are likely to affect lifetime health-care costs, nor indirect costs of obesity such as reduced productivity. Nevertheless, these findings suggest that although effective obesity prevention reduces the costs of obesity-related diseases, this reduction is offset by the increased costs of diseases unrelated to obesity that occur during the extra years of life gained by slimming down.

**Additional Information.:**

Please access these Web sites via the online version of this summary at http://dx.doi.org/doi:10.1371/journal.pmed.0050029.

- The MedlinePlus encyclopedia has a page on obesity (in English and Spanish)
- The US Centers for Disease Control and Prevention provides information on all aspects of obesity (in English and Spanish)
- The UK National Health Service's health Web site (NHS Direct) provides information about obesity
- The International Obesity Taskforce provides information about preventing obesity
- The UK Foods Standards Agency, the United States Department of Agriculture, and Shaping America's Health all provide useful advice about healthy eating
- The Netherlands National Institute for Public Health and the Environment (RIVM) Web site provides more information on the cost of illness and illness prevention in the Netherlands (in English and Dutch)

## Introduction

Because obesity is acknowledged as a major cause of morbidity and mortality [1,2], prevention of obesity is a target of health policy in many countries [3,4]. At the same time, many countries struggle to control ever-increasing health-care expenditures. The Organization for Economic Cooperation and Development suggested in 2005 that both goals could be achieved simultaneously, since *“well-designed public health programmes may contribute to the prevention of illness and help relieve some of the cost pressures on health care systems”* [5]. Such a promise of better health equaling lower costs is not new [6], yet is debatable. In fact, for smoking it has been argued that successful prevention will in the end increase expenditure exactly because it is successful [7,8]. The explanation for this hypothesis is that the life-years gained by prevention are not all lived in full health. While effective prevention will lead to a decrease in risk factor-related diseases, which may result in savings, these savings may be offset by cost increases related to an increase in diseases in life-years gained. Therefore, prevention may induce more health-care costs in the long run than it saves in the short run. Whether this possibility is true, however, will strongly depend on the risk factor concerned. An important determinant is whether this risk factor primarily causes relatively cheap lethal diseases or rather expensive chronic ones [9]. Since the diseases associated with obesity differ from those associated with smoking it is worthwhile to investigate whether or not prevention of obesity might indeed, as is sometimes suggested, relieve financial pressures on health-care systems. If it does not, of course, it does not imply that preventing obesity is not worthwhile, since the associated health gain is valuable in itself, for society and the individuals concerned.

In recent years several estimates of health-care costs attributable to obesity have been published [4,10–20]. Not only do such estimates vary enormously because of differences in methodology and definitions of health-care costs, these studies do not take into account the additional costs of “substitute” diseases that might occur during life-years gained. To our knowledge only two studies used the appropriate lifetime perspective [19,20], while only one [20] took into account medical costs of substitute diseases in life-years gained. It concluded that obesity causes higher lifetime medical costs, implying that prevention in this area can indeed result in cost savings.

In this study we present new estimates of annual and lifetime health-care costs of obesity in The Netherlands, and make comparisons between cohorts of people with different patterns of morbidity and mortality—namely, on the one hand smokers and on the other “healthy-living” people. This comparison provides two clear reference points for the case of obesity. A cohort approach was chosen to avoid blurring the comparison by demographic heterogeneity and to allow for a lifetime perspective. We included both the costs of diseases directly associated with obesity and smoking and those of other diseases that tend to occur as life-years are gained.

## Methods

To estimate annual and lifetime health-care costs conditional on the presence of risk factors, the National Institute for Public Health and the Environment chronic disease model (RIVM-CDM) was used. The RIVM-CDM is a dynamic population model that describes the life course of cohorts in terms of transitions between risk factor classes and changes between disease states over time. Smoking classes distinguished in the model are never-smokers, current smokers, and former smokers. Body weight is modeled in three classes using body mass index (BMI) as an indicator: 18.5 ≤ BMI < 25 (normal weight), 25 ≤ BMI < 30 (overweight), BMI ≥ 30 (obese). The RIVM-CDM has been used in disease projections and cost effectiveness analyses [21–25]. With the model we estimated survivor numbers and disease prevalence numbers for three different hypothetical cohorts consisting of 500 men and 500 women aged 20 y at baseline: (1) an “obese” cohort, never-smoking men and women aged 20 with a BMI above 30; (2) a “healthy-living” cohort, never-smoking men and women aged 20 with normal weight (18.5 ≤ BMI < 25); and (3) a “smoking” cohort, men and women aged 20 with normal weight who had smoked throughout their life.

Cohorts were simulated until everybody in the cohort had died. The methods and input data we used to estimate survivor and disease prevalence numbers for the different cohorts with the model were discussed in depth elsewhere [26] (see also Table S1 and Texts S1 and S2 for more information on the RIVM-CDM). In short, risk factors were linked to 22 obesity- and/or smoking-related chronic diseases through relative risks of disease incidence for each risk factor level, to model the chain leading from risk factor to disease to death. In addition, risk factor levels influence mortality directly through mortality from diseases that are not explicitly modeled. The diseases modeled account for roughly 60% of total morbidity [27] and mortality, and 15% of total health-care costs in The Netherlands [28]. The RIVM-CDM is programmed as a deterministic Markov model, i.e., the simulation model calculates the expected outcomes in one run. Therefore, more replications would not improve the results, which differs from a so-called microsimulation or Monte Carlo simulation model. We chose 500 men and 500 women purely for convenience.

No ethics committee approval was required for this study.

Cost of illness (COI) data from The Netherlands for 2003 were used to estimate health-care expenditure for the different cohorts [28]. The 2003 COI study was a sequel to earlier COI 1999 studies in the Netherlands [29–31] and COI estimates were made using the health-care cost definitions of the System of Health Accounts (SHA) for reasons of international comparability of costs [32]. Average annual costs per patient having a certain disease were calculated by dividing total annual costs by Dutch prevalence numbers for each disease in 2003. Health-care costs for the different cohorts were then calculated as follows. First, the annual disease costs per patient were multiplied by RIVM-CDM projections of future prevalence numbers for each chronic disease in the model. Then, to calculate health-care costs for all “other” diseases, the numbers of survivors were multiplied by age- and sex-specific cost profiles of “remaining” costs. These latter are the difference between total health-care costs and the costs of the diseases incorporated explicitly in the model. These costs include, for instance, the costs of mental and behavioral disorders. Finally, these two categories of costs, one related and the other unrelated to the risk factor under study, were added to estimate annual costs. To calculate lifetime health-care costs of the three different cohorts [33], annual costs were added over time. To reflect the concept of time preference, meaning that an amount of money spent or saved in the future is worth less than the same amount today, net present values were calculated using discount rates of 3% and 4%. Using the differences in lifetime health-care costs compared to the healthy-living cohort we calculated whether or not avoidance of obesity and smoking resulted in lower health-care costs.

To investigate the robustness of our results with respect to future changes in disease epidemiology and health-care costs, and different definitions of health-care costs, a series of sensitivity analyses were performed by estimating the lifetime health-care costs in different scenarios:

### 

#### Scenario 1.

Assumes a yearly decrease of 1% in the incidence and mortality rates for all diseases included in the model. This is roughly the same yearly decrease as was used in the Global Burden of Disease projections of global mortality and burden of disease [34].

#### Scenario 2.

Assumes a yearly decrease in all relative risks of the obese and smoking cohort to reflect selective disease prevention efforts in smokers and obese as has been observed in the past [35,36]. This was done using the following formula: RR(t) = (RR(t − 1) − 1} × 0.99 + 1 where RR(t) is the relative risk in year t.

#### Scenario 3.

Assumes a yearly increase of 1% in health-care costs for all diseases per person.

#### Scenario 4.

Adopts a broader definition of health-care costs (like the one commonly used in The Netherlands [37]), which includes a broader range of long-term and residential care than in the SHA as used in baseline estimates, which is especially relevant in case of increased longevity.

#### Scenario 5.

Adopts a narrower definition of health-care costs by excluding all expenditure on nursing and residential care mentioned in the SHA definition. These costs can cause substantial variation in cost-of-illness estimations between countries [37]. The narrower set of costs improves the international comparability of the figures presented.

#### Scenario 6.

Uses relative mortality risk estimates for persons with 30 ≤ BMI < 35, published by Flegal et al. [38] as input for the simulation model for the obese cohort. Mortality estimates vary substantially as a function of BMI in the higher ranges beyond the cutoff BMI value of 30. Lumping together all values above 30 into one category masks this significant variation in mortality and thus also in lifetime health-care costs, possibly leading to biased estimates. In fact, there still is scientific debate about the exact values of the mortality risks associated with different levels of BMI. The article by Flegal et al. [38] attracted much attention because their estimates of the excess mortality associated with obesity were much lower than previously thought.

#### Scenario 7.

Uses relative mortality risk estimates for persons with a BMI ≥ 35, published by Flegal et al. [38] as input for the simulation model for the obese cohort.

## Results

Table 1 shows remaining life expectancy and the lifetime health-care costs for the three cohorts, specified by disease category.

**Table 1:**
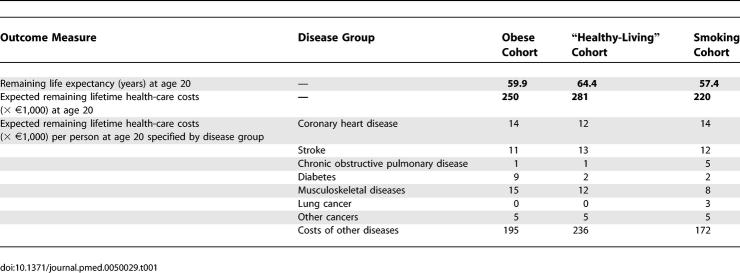
Life Expectancy (Years) and Expected Lifetime Health-Care Costs per Capita (Price Level 2003 × €1,000) at 20 Years of Age for the Three Cohorts

The obese cohort has the highest health-care costs for diabetes and musculoskeletal diseases compared to the other cohorts. Lifetime costs for cancers other than lung cancer are equal for all cohorts. Despite differences in life expectancy, the costs for stroke are similar for all cohorts. The most pronounced difference in costs occurs in the category “costs of other diseases,” which is purely the result of different life expectancies.

**Figure 1:**
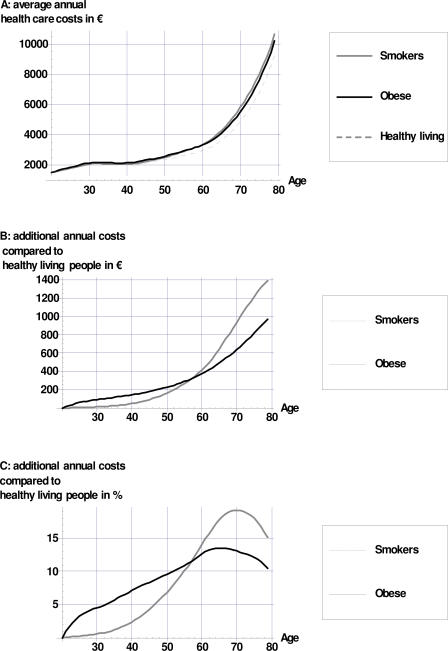
Average Additional Annual per Capita Costs for Smoking and Obese Individuals Compared to “Healthy-Living” Individuals

Figure 1 displays average annual health-care costs per healthy-living person, smoker, and obese person. At all ages, smokers and obese people incur more costs than do healthy-living persons. Until age 56, average annual health-care costs are highest for an obese person. In higher age groups smokers are more expensive.

Despite the higher annual costs of the obese and smoking cohorts, the healthy-living cohort incurs highest lifetime costs, due to its higher life expectancy, as shown in Table 1. Furthermore, the greatest differences in health-care costs are not caused by smoking- and obesity-related diseases, but by the other, unrelated, diseases that occur as life-years are gained (Table 1). Therefore, successful prevention of obesity and smoking would result in lower health-care costs in the short run (assuming no costs of prevention), but in the long run they would result in higher costs.

**Figure 2:**
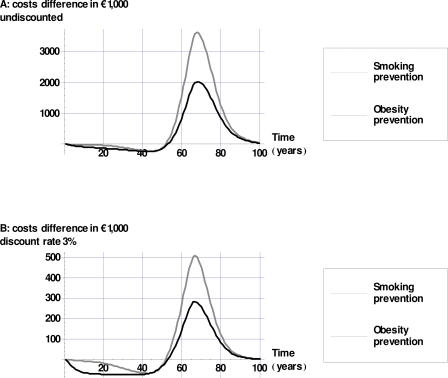
Differences in Aggregated Health-Care Costs over Time if Successful Prevention Converts the Obese and Smoking Cohort into a Healthy-Living Cohort (Undiscounted and with a Discount Rate of 3%)

To zoom in on what might happen to health-care costs if successful prevention converts the obese and smoking cohort to a healthy-living cohort, Figure 2 displays the differences in total health-care costs over time between the obese and smoking cohorts compared to the healthy-living cohort. Figure 2 shows that in approximately the first 50 years after the hypothetical lifestyle change of the cohort, cost savings are realized through the reduced incidence of smoking- and high-BMI–related diseases. After this period, additional health-care costs occur during life-years gained. The initial savings are higher for the converted obese cohort, primarily the result of savings due to a reduced incidence of diabetes and of nonlethal diseases such as osteoarthritis and lower-back pain. Furthermore, Figure 2 demonstrates that the initial savings weigh more heavily than do additional costs in the long run if costs are discounted. Cumulative differences in health-care costs are lower for obesity prevention than for smoking prevention: at discount rates of, respectively, 3% and 4% successful smoking prevention would result in additional health-care costs of €7.1 and €3.4 million (assuming costless intervention). For obesity prevention these figures would amount to €1.8 and €1.0 million. Only for discount rates above 4.7% would costless obesity prevention be cost saving. For smoking prevention to be cost saving, the discount rate for costs should be at least 5.7%

Table 2 displays the results of the sensitivity analyses. Expected health-care costs for all cohorts, and relative differences between cohorts increase in scenario 1 (decreasing incidence and mortality rates) due to increases in life expectancy. In scenario 2 (decreasing relative risks), differences between the cohorts become less pronounced. In scenario 3 (increasing health-care costs) absolute estimates of lifetime health-care costs and differences between the cohorts increase. This is due to the fact that the yearly increase in health-care costs will be mostly felt at older ages. Under the broader Dutch definition of health-care costs (scenario 4), differences between cohorts increase. Excluding costs of nursing homes (scenario 5) attenuates the differences between cohorts. Estimates of lifetime health-care costs using lower relative mortality risks for the obese cohort as input narrow the differences between the obese and healthy-living cohort (scenarios 6 and 7). The rank order of lifetime health-care costs for the cohorts, however, is the same in all scenarios.

**Table 2:**
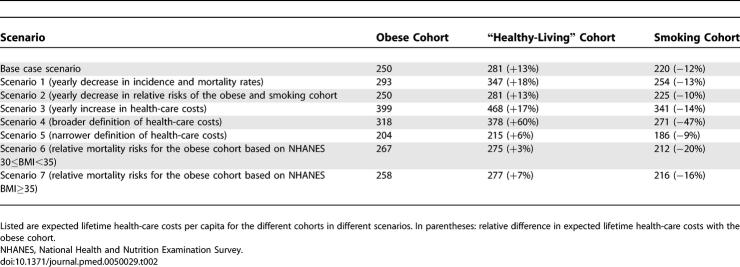
Results of Sensitivity Analyses

## Discussion

In this study we have shown that, although obese people induce high medical costs during their lives, their lifetime health-care costs are lower than those of healthy-living people but higher than those of smokers. Obesity increases the risk of diseases such as diabetes and coronary heart disease, thereby increasing health-care utilization but decreasing life expectancy. Successful prevention of obesity, in turn, increases life expectancy. Unfortunately, these life-years gained are not lived in full health and come at a price: people suffer from other diseases, which increases health-care costs. Obesity prevention, just like smoking prevention, will not stem the tide of increasing health-care expenditures. The underlying mechanism is that there is a substitution of inexpensive, lethal diseases toward less lethal, and therefore more costly, diseases [9]. As smoking is in particular related to lethal (and relatively inexpensive) diseases, the ratio of cost savings from a reduced incidence of risk factor–related diseases to the medical costs in life-years gained is more favorable for obesity prevention than for smoking prevention.

The simulation model we used to study the lifetime medical costs associated with obesity employs data and assumptions similar to those used to calculate so-called “attributable fractions” [39] that serve to determine which proportion of health care may be attributed to particular risk factors [10]. The main difference between the two approaches is that our model can take into account differences in life expectancy. Using the same simulation model and methodology employed in this paper we calculated that 2.0% of total health-care costs in The Netherlands in 2003 could be attributed to overweight (BMI > 25). For smoking, this percentage equaled 3.7%. Given differences in overweight and smoking prevalence between the US and The Netherlands, these figures compare well with previous research [4,8,10–18]. With respect to lifetime medical costs for smokers, our results are in line with other studies that used a lifetime perspective. Barendregt et al. [7], using Dutch data, and Sloane et al. [8], in the US health-care setting, both found that the high medical costs of smoking-related diseases are more than offset by lower survival of smokers. For costs attributable to obesity, only one previous study used a similar methodology, employing a lifetime perspective and including all medical costs. In contrast to our analysis, it concluded that obesity increases lifetime medical costs [20]. This discrepancy may be explained in several ways. First, Allison and colleagues truncated their analysis at age 85, which—even assuming no differences in mortality between cohorts after this age—biases the results toward relatively higher lifetime medical costs attributable to obesity. Second, they based their estimates on another study in which the costs attributable to obesity were not stratified by age group [10]; to compensate for this lack of information they hypothesized an age gradient. Third, their age-related costs increased more gradually, which may be due to a narrower definition of health-care costs. Fourth, it could well be the case that our cost definition was broader and included more so-called costs of care instead of cure, which are related to age rather than to disease per se.

Some aspects of our study methodology need to be emphasized. First, in the simulation model employed, disease incidence rates are coupled to risk factor levels. Linking costs per disease to the estimated disease prevalence over time then allows for an explicit causal link between BMI status and health-care costs. This is an important point, since in studies using individual-level data comprising both BMI and health-care use, the causality of the relationship between BMI and health-care use is usually left unspecified [11,15,40]. As a result, the observed differences between the groups might have been associated with confounding variables, e.g., socioeconomic status.

Second, the health-care costs employed in the model were a function of age and disease status but not of proximity to death, which has been proposed as an important determinant of health-care costs [41–43]. However, by modeling most primary causes of death (coronary heart disease, stroke, and different types of cancers) in our example, we implicitly have taken into account “time to death” as an important explanatory variable of health-care costs, since postponement of these lethal diseases through prevention also postpones the costs of these diseases.

Third, we assumed that costs per patient for each risk factor–related disease are equal, irrespective of risk factor status. This similarity might not always be the case. For instance, treatment costs of lower-back pain could depend on BMI status.

Fourth, it is important to stress that we have focused solely on health-care costs related to smoking and obesity, ignoring broader cost categories and consequences of these risk factors to society. It is likely, however, that these impacts will be substantial. For instance, reduced morbidity in people of working age may improve productivity and thus result in sizeable productivity gains in society (e.g., [44]). In the case of smoking and obesity, these indirect costs could well be higher than the direct medical costs [8,18]. Moreover, from a societal perspective, other potentially substantial costs and consequences need to be considered, such as those related to informal care, the damage due to fires caused by smoking, or the reduced well-being of family members due to morbidity and premature death. These different cost categories emphasize the influence the perspective taken in economic analyses has on the conclusions. From a welfare economic perspective the societal perspective is, in fact, the most relevant [45], although in practice many evaluations take a narrower perspective, which more closely conforms to the perspective most relevant to the decision-maker they are trying to inform [46].

Fifth, in our simulation model there are no gradations of obesity, which are critical to relative risks, mortality impact, and thus also to lifetime health-care costs. However, our sensitivity analyses revealed that even if mortality risks for the obese group were based solely on the group 30 ≤ BMI < 35, lifetime health-care costs of the obese would still be lower than those of healthy-living persons.

Finally, we assumed that no transitions occur between risk factor classes over time. In reality, of course, transitions between classes do occur: some smokers quit (and some of them might start again later) and obese people of course might lose and regain weight over time.

A remaining and most important question is whether prevention should be cost-saving in order to be attractive. Obviously, the answer is that it need not be cost-saving: like other forms of care it “merely” needs to be cost-effective. Bonneux et al. [9] made this very clear: *“The aim of health care is not to save money but to save people from preventable suffering and death. Any potential savings on health care costs would be icing on the cake.”* If prevention can bring additional health to a population at relatively low costs, it is a good candidate for funding [47]. However, the present study demonstrates that sound estimates of medical costs in life-years gained should be taken into account in cost-effectiveness analysis of prevention. In this respect it is interesting to note that in the area of smoking cessation and weight loss, favorable cost-effectiveness results have been shown even if medical costs in life-years gained are taken into account [22,26,33]. Prevention may therefore not be a cure for increasing expenditures—instead it may well be a cost-effective cure for much morbidity and mortality and, importantly, contribute to the health of nations.

## Supporting Information

Table S1 Input Data of the RIVM Chronic Disease Model Used in the Calculations (335 KB XLS)

Text S1 Details on the Structure of the RIVM Chronic Disease Model (106 KB PDF)

Text S2 Additional Results Specified by Disease (141 KB PDF)

## Abbreviations

BMI: body mass index
COI: cost of illness
RIVM-CDM: National Institute for Public Health and the Environment chronic disease model
SHA: System of Health Accounts
