# Supplementary material for: Lifetime Medical Costs of Obesity: Prevention No Cure for Increasing Health Expenditure
Source: PLoS Med. 2008 Feb 5;5(2):e29. doi: 10.1371/journal.pmed.0050029 (PMC2225430; doi:10.1371/journal.pmed.0050029)
Supplement: Supplementary file 1 [file pmed.0050029.sd001.pdf]

Appendix A: RIVM Chronic Disease Model

Introduction

The RIVM Chronic Disease Model (CDM) is a state transition Markov-type simulation model that describes how morbidity and mortality for several chronic diseases change over time in the Dutch population as a result of changes in epidemiological risk factors [1 2]. In general, the state transition model is a suitable and accepted model to describe demographic / epidemiological processes [3]. Disease experts and modelers have cooperated in building and testing of the CDM and several studies with different applications of the model have already been published [4-10]. The CDM has been formulated as a set of time-continuous differential and is implemented in the software package Mathemetica.

Basic structure

In the CDM different states are defined for the risk factor classes (e.g. never smokers, current smokers and former smokers) and states for the diseases included in the model (e.g. stroke: yes or no). In the starting year of the simulation period all persons are distributed over these states. Then, in time steps of 1-year, persons move from one state to another. These transitions are governed by so-called transition rates. E.g., class transition rates between the BMI states 'normal weight' and 'overweight' govern the change of the BMI distribution in the population, incidence rates between the states 'without diabetes' and 'with diabetes' govern the disease prevalence rates, and mortality rates from the state 'alive' to 'deceased' govern the surviving population numbers. For our calculations, we did not take into account transitions between risk factor classes over time. Thus, all cohorts are closed in the sense that no transitions occur between risk factor classes over the life-time. The transition rate is assumed independent from the preceding states and depends only on the present state defined by risk factor class, disease state, sex and age. The disease incidence rates depend on the risk factor class, using relative risk values. E.g., 'overweight' persons have higher diabetes risks than persons with 'normal weight'. For non diseased, the mortality rates depend on risk factor class, e.g., obese persons have higher mortality risks than persons with a normal weight. The mortality rates also depend on the disease states, but are conditional hereon independent on the risk factor values. E.g., the excess mortality risks of people with diabetes compared to people without diabetes are equal for all BMI states.

We assumed that all risk factors that are distinguished are independently distributed, e.g. we assumed that the distribution of smoking independent from BMI. All disease incidence risks

were made dependent on these risk factors by multiplying the baseline risk with relative risk values specified by risk factor class. Moreover, we assumed for some disease pairs independent effects of one disease on the other. E.g., people with diabetes have higher risks of myocardial infarction compared to people without diabetes, independently from overweight and the other risk factors.

The main model outcome variables are incidence, prevalence and mortality numbers, specified by disease, age and gender. For the calculation of lifetime health care costs of the different cohort we used the number of survivors and disease prevalence numbers of the different cohorts.

Input data

Smoking classes distinguished in the CDM are never smokers, current smokers and former smokers. Body weight is modeled in three classes using Body Mass Index (BMI) as indicator: 18.5<BMI<25 (normal weight), 25≤BMI< 30 (overweight), BMI ≥30 (obesity). Table A1 displays the diseases modeled in the CDM that are related to BMI and/or smoking.

Table 1: diseases modeled in the CDM and their relation to smoking and obesity

	Related to smoking	Related to obesity
**Cardiovascular disease**		
Acute myocardial infarct (AMI)	+	+
Angina pectoris	+	+
Chronic Heart Failure	+	+
Stroke (CVA)	+	+
**Cancer**		
Lung	+	
Stomach	+	
Oesophagus	+	
Pancreas	+	
Oral cavity	+	
Larynx	+	
Uriny bladder	+	
Kidney	+	+
Rectum		+
Colon		+
Breast		+
Prostate		+
Endometrium		+
**Other**		
COPD	+	
Diabetes	+	+
Atrhrosis of the hip		+
Arthrosis of the knee		+
Dorsopathies (low back pain)		+

We included those diseases in the model, that are related to obesity according to current scientific consensus [11]. For the selection of smoking related diseases we followed the report by the Surgeons' General [12]. For all diseases related to smoking and/or obesity modeled in the CDM, age and sex specific incidence, prevalence and mortality rates were estimated using a three state transition model [13-15]. The higher mortality risks of patients compared to disease-free persons were calculated from published relative survival proportions for types of cancer, and from incidence and prevalence figures for other chronic diseases, using an Incidence, Prevalence, Mortality (IPM) model[16]. Data on risk factor class prevalence were obtained from representative national or regional surveys Risk factor prevalence rates for smoking are based on data of STIVORO [17]. For obesity, data from the annual POLS survey from Statistics Netherlands are used[18]. Relative risks on morbidity and mortality for smoking and obesity are based on several observational studies [19-65]. Relative risks of the three BMI classes were calculated in three steps. First, a quadratic function was estimated to describe the non-linear relation between BMI and all cause mortality relative risks for different studies. The parameters of these functions were then plotted against age to estimate an age gradient. In a third step, average relative risks for the three different BMI classes were computed using the BMI distribution within these classes in the Netherlands. For the current and former smoking classes distinguished in the CDM, data were used from studies that reported relative risks for all current and/or all former smokers specified by gender and age. To estimate health care costs for the different cohorts data of the Costs of Illness in the Netherlands study were used [66]. In that study the total direct health care costs in the Netherlands of 2003 are uniquely attributed to disease categories specified by gender and age classes. All input data were specified by gender and age (see Appendix B).

Mathematical model equations

The model consists of three parts. In the model initialization part the parameter values and the initial distribution of the population over all model states are calculated. In the model simulation part the 1-year changes of the prevalence numbers are calculated. These changes are the result of transitions between risk factor classes and disease states[a]. The transition numbers are computed as the transition rates times the prevalence numbers at the start of the 1-year time-interval. Finally, in the model post-processing part the values of the model output variables such as health care costs and life expectancy. are calculated from the results of the simulation part. The Runge-Kutta method is used to find initial values and numerical approximations for 1 year time steps used in the CDM.

[a] Since, in this application, we did not take into account transitions between risk factor classes we will focus exclusively on the transitions between disease states.

Model initialization part

The parameters calculated here are the baseline disease incidence rates, i.e. the incidence rate values for normal weight never smokers, the mortality rates for other causes of death.

Mortality rates from Statistics Netherlands for the year 2004 are attributed to risk factor classes to derive mortality rates specified by risk factor class. Assuming independence between risk factor class prevalence rates and multiplicative relative risks (i.e. no interaction on log-linear scale) we can write mortality rates for the different cohorts as (for notational simplicity, age and sex indices have been omitted in the notation throughout the paper):

$$m(tot \mid s_j, b_k) = m(tot)_0 * RR(tot \mid s_j) * RR(tot \mid b_k) \qquad (1)$$

$m(tot \mid s_i, b_j)$	*all cause mortality rate for cohort for smoking class j BMI class k*
$m(tot)_0$	*baseline all cause mortality rate for 'healthy living' cohort*
$RR(tot \mid s_j)$	*relative risk all cause mortality smoking class j*
$RR(tot \mid b_k)$	*relative risk all cause mortality BMI class k*

Using (1) we can write the baseline mortality rate for the 'healthy living' cohort as:

$$m(tot)_0 = \frac{m(tot)}{\sum_{j,k} RR(tot \mid s_j) * RR(tot \mid b_k) * s_j * b_k} \qquad (2)$$

m(tot)	*all cause mortality rate (Statistics Netherlands)*
s_j	*prevalence rate smoking class j*
b_k	*prevalence rate BMI class k*

Baseline disease incidence rates and risk factor class specific disease incidence rates are calculated in the same fashion as mortality rates:

$$i(d \mid s_j, b_k) = i(d)_0 * RR(d \mid s_j) * RR(d \mid b_k) \qquad (3)$$

$i(d \mid s_i, b_j)$	*incidence rate disease d for cohort smoking class j BMI class k*
$i(d)_0$	*baseline incidence rate for 'healthy living' cohort*
$RR(d \mid s_j)$	*relative risk for disease d smoking class j*
$RR(d \mid b_k)$	*relative risk for disease d BMI class k*

$$i(d)_0 = \frac{i(d)}{\sum_{j,k} RR(d \mid s_j) * RR(d \mid b_k) * s_j * b_k} \tag{4}$$

i(d) *population incidence rate disease d*

The CDM describes disease prevalence numbers for each disease separately and it is assumed that the disease-specific attributed mortality rates are additive. The all cause mortality rates are the sum of the disease specific attributed mortality rates and the mortality rates from other causes of death:

$$m(oc) = m(tot) - \sum_{d} am(d)\, p(d) \tag{5}$$

m(oc) *mortality rate for other causes of death*
p(d) *disease d prevalence rates (several sources)*
am(d) *mortality rate attributed to disease d*

Mortality rates attributed to diseases are calculated by dividing the cause specific mortality rates registered by Statistics Netherlands by disease specific prevalence rates:

$$am(d) = \frac{c(d)}{p(d)} \tag{6}$$

c(d) *cause specific mortality rate disease d*

It is assumed that for any disease the attributed mortality is independent from the risk factor levels. This means that the risk factors affect the disease prognosis only through increased

risks for other diseases and mortality from other causes of death. Using the relative risk for the incidence of diseases as an approximation for relative risk for the prevalence of diseases we calculated the relative risks for other causes of death:

$$RR(oc \mid s_j) = \frac{RR(tot \mid s_j) * m(tot)_{0S} - \sum_d RR(d \mid s_j) * am(d) * p(d)_{0S}}{m(oc)_{0S}} \tag{7}$$

$$p(d)_{0S} = \frac{p(d)}{\sum_j RR(d \mid s_j) * s_j} \tag{8}$$

$$m(oc)_{0S} = \frac{m(oc)}{\sum_j RR(oc \mid s_j) * s_j} \tag{9}$$

$$m(tot)_{0S} = \frac{m(tot)}{\sum_j RR(tot \mid s_j) * s_j}$$

$RR(oc \mid s_j)$	*relative risk for other cause mortality smoking class j*
$m(oc)_{0S}$	*baseline other cause mortality rate for non smoking cohort*
$p(d)_{0S}$	*baseline prevalence rate disease d for non smoking cohort*
$m(tot)_{0S}$	*baseline all cause mortality rate for non smoking cohort*

These equations can be solved for $RR(oc \mid s_{j,})$ by substituting equations (8) and (9) into equation (7). In a similar fashion, relative risks for other causes mortality of for overweight and obesity can be derived. Given $RR(oc \mid s_{j,})$ and $RR(oc \mid b_{k,})$ the baseline other cause mortality rate can be found:

$$RR(oc \mid s_j, b_k) = RR(oc \mid s_j) * RR(oc \mid b_k) \tag{10}$$

$$m(oc)_0 = \frac{m(oc)}{\sum_{j,k} RR(oc \mid s_j) * RR(oc \mid b_k) * s_j * b_k} \tag{11}$$

$RR(oc \mid s_j, b_k)$	*relative risk for other cause mortality smoking class j BMI class k*
$RR(oc \mid b_k)$	*relative risk for other cause mortality BMI class k*
$m(oc)_0$	*baseline other cause mortality rate for 'healthy living' cohort*

$p(d)_0$	*baseline prevalence rate disease d for 'healthy living' cohort*

Model simulation part

Risk factors and diseases are linked through relative risks of disease incidence for each risk factor. That is, incidence rates for each risk factor class are found as relative risks times baseline incidence rate. The general assumption used is that conditional on the risk factors included, the disease event rates are independent. For the 'healthy living cohort' relative risks equal one. Formula (12) denotes the change over time in the prevalence rate of disease *d* for a cohort, homogeneous in its risk factor class prevalence, as a function of relative risks, incidence and mortality rates:

$$\frac{dp(d \mid t)}{dt} = \left(i(d)_0 * RR(d \mid s_j) * RR(d \mid b_k) - em(d) * p(d \mid t)\right) * \left(1 - p(d \mid t)\right) \qquad (12)$$

$p(d \mid t)$	*prevalence rate disease d at time t*
$i(d)_0$	*baseline incidence rate disease d for 'healthy living' cohort*
$RR(d \mid s_j)$	*relative risk for disease d for smoking class j*
$RR(d \mid b_k)$	*relative risk for disease d for BMI class k*
$em(d)$	*excess mortality rate disease d*

The CDM describes disease prevalence numbers for each disease separately and it is assumed that the disease-specific attributed mortality rates are additive. Given the relations between disease specific attributed mortality, other causes mortality, disease prevalence rates and relative risks we can describe the change in population numbers needed to estimate life expectancy:

$$\frac{dN(t)}{dt} = RR(oc \mid s_j) * RR(oc \mid b_k) * m(oc)_0 * N(t) - \sum_d am(d) * p(d \mid t) * N(t) \qquad (13)$$

$RR(oc \mid s_j)$	*relative risk for other causes mortality smoking class j*
$RR(oc \mid b_k)$	*relative risk for other causes mortality BMI class k*
$m(oc)_0$	*baseline other causes mortality rate for 'healthy living' cohort*
am(d)	*mortality rate attributed to disease d*

The difference of the mortality rates for persons with and without the disease can be interpreted as the excess mortality rate for that disease. However, in a model with multiple diseases these excess mortality rates cannot be interpreted as mortality uniquely attributable to a disease, since the excess mortality rates can also be caused by other co-morbid chronic diseases, e.g. coronary heart disease being a complication of diabetes. Therefore, in the calculation of the prevalence rates excess mortality rates are used, while in the calculation of the number of survivors disease specific attributed mortality rates are used.

Postprocessing

Calculating life expectancy

Equation (14) displays the formula with which we estimated remaining life expectancy for the different cohorts:

$$LE = \frac{\sum_t n(t)}{n(0)} \qquad (14)$$

LE	*Life Expectancy*
n(t)	*number of survivors of the cohort at time t*
n(0)	*initial size of the cohort at time 0*

Calculating health care costs

To assign the Costs of Illness data that give total costs per BMI or smoking related disease to costs of individual patients, total costs per disease were divided by the disease prevalence numbers for 2003:

$$cp(d) = \frac{COI(d)}{p(d)} \qquad (15)$$

cp(d) *annual costs per patient having smoking and/or BMI related disease d*

COI(d) *total costs disease d*

Given the estimated annual costs per disease per patient, health care costs are then simply the product of these costs times the prevalence numbers for that disease estimated with the CDM:

$$hc(d \mid t) = cp(d) * p(d \mid t) * n(t) \qquad (16)$$

hc(d|t) *health care costs disease d at time t*

To calculate health care costs for all other diseases that are unrelated to smoking and/or BMI the numbers of survivors were multiplied by age and sex specific average health care costs for other diseases (i.e. costs remaining when costs of causally related diseases are subtracted from total costs):

$$co = ac - \frac{\sum_d COI}{pop} \qquad (17)$$

co *annual health care costs per person for all other diseases*
ac *average annual health care costs per person*

Since the COI study is a top down study in which health care costs are uniquely attributed to diseases we assume that health care costs for patients with more than one CDM disease equals the sum of the disease costs for these individual diseases. The resulting equation for estimating lifetime health care costs then becomes:

$$lhc = \sum_t n(t) * \{co + \sum_d p(d \mid t) * cp(d)\} \qquad (18)$$

lhc *lifetime health care costs*

References

1. Hoogenveen RT , de Hollander AEM, van Genugten MLL. The chronic disease modelling approach. 1998; RIVM Rapport 266750001.

2. Hoogenveen R, Feenstra T, Baal Pv, Baan C. A conceptual framework for budget allocation in the RIVM Chronic Disease Model - A case study of Diabetes Mellitus . national Institute of Public Health and the Environment (RIVM), 2005; RIVM rapport 260706001 .

3. Manton K, Stallard E. Chronic disease modelling: measurement and evaluation of the risks of chronic disease processes. New York: Oxford University Press.

4. Bemelmans W, van Baal P, Wendel-Vos W *et al*. The costs, effects and cost-

effectiveness of counteracting overweight on a population level. A scientific base for policy targets for the Dutch national plan for action. Prev Med 2007.

5. Jacobs-van der Bruggen MA, Bos G, Bemelmans WJ, Hoogenveen RT, Vijgen SM, Baan CA. Lifestyle Interventions Are Cost-Effective in People With Different Levels of Diabetes Risk: Results from a modeling study. Diabetes Care 2007; 30(1):128-34.

6. Feenstra TL, Hamberg-van Reenen HH, Hoogenveen RT, Rutten-van Molken MP. Cost-effectiveness of face-to-face smoking cessation interventions: a dynamic modeling study. Value Health 2005; 8(3):178-90.

7. Feenstra TL, van Genugten ML, Hoogenveen RT, Wouters EF, Rutten-van Molken MP. The impact of aging and smoking on the future burden of chronic obstructive pulmonary disease: a model analysis in the Netherlands. Am J Respir Crit Care Med 2001; 164(4):590-6.

8. Struijs JN, van Genugten ML, Evers SM, Ament AJ, Baan CA, van den Bos GA. Modeling the future burden of stroke in The Netherlands: impact of aging, smoking, and hypertension. Stroke 2005; 36(8):1648-55.

9. Vijgen S, van Baal P, Hoogenveen R, de Wit G, Feenstra T. Cost-effectiveness analyses of health promotion programs: a case study of smoking prevention and cessation among Dutch students. Health Educ Res 2007.

10. van Baal PH, Brouwer WB, Hoogenveen RT, Feenstra TL. Increasing tobacco taxes: a cheap tool to increase public health. Health Policy 2007; 82(2):142-52.

11. National Audit Office. Tackling obesity in England. Report by the comptroller and auditor general. London: NAO.

12. US Department of Health and human services PHSCfDCCfCDPaHP. Reducing Tobacco Use: A Report of the Surgeon General. 2000.

13. Hoogenveen RT, Gijsen R., van Genugten MLL, Kommer GJ, Schouten JSAG, de Hollander AEM. Dutch DisMod. Constructing a set of consistent data for chronic disease modelling. Bilthoven: National Institute of Public Health and the Environment (RIVM) 2000.

14. Hoogenveen RT, Gijsen R. Dutch DisMod for several types of cancer. Bilthoven: National Institute of Public Health and the Environment (RIVM) 2000.

15. Barendregt JJ, Van Oortmarssen GJ, Vos T, Murray CJ. A generic model for the assessment of disease epidemiology: the computational basis of DisMod II. Popul Health Metr 2003; 1(1):4.

16. Barendregt JJ, Van Oortmarssen GJ, Vos T, Murray CJ. A generic model for the assessment of disease epidemiology: the computational basis of DisMod II. Popul Health Metr 2003; 1(1):4.

17. Annual Report 2004. Den Haag: STIVORO, Dutch Foundation for Smoking and Health .

18. Statistics Netherlands . Statline [www.cbs.nl] [Web Page]. (Accessed 14 October 2003).

19. Wannamethee SG SAWPWM. Role of risk factors for major coronary heart disease events with increasing length of follow up. Heart 1999; 81(4):374-9.

20. Colditz G.A., Bonita R., Stampfer M.J. *et al*. Cigarette smoking and risk of stroke in middle-aged women. NEJM 1988; 318(15):937-41.

21. Robbins A.S., Manson J.E., Lee I-M., Satterfield Z., Hennekens C.H. Cigarette smoking and stroke in a cohort of U.S. male physicians. Ann Intern Med 1994; 120:458-62.

22. Tverdal A., Thelle D., Stensvold I., Leren P., Bjartveit K. Mortality in relation to smoking history: 13 years'follow-up of 68,000 Norwegian men and women 35-49 years. J Clin Epid 1993; 46(5):475-87.

23. Slattery ML SWMKMMSCLTNS. CYP1A1, cigarette smoking, and colon and rectal cancer. 160. 2004:842-52.

24. Viswanathan AN FDDVIHDBRRBCGHSE. Smoking and the risk of endometrial cancer: Results from the Nurses' Health Study. 114. 2005:996-1001.

25. Zhang B FRCJBSAMRJJMRTMA. Smoking Cessation and Lung Cancer Mortality in a Cohort of Middle-aged Canadian Women. 15. 2005:302-9.

26. Zeegers MP TFDEvDBPA. The impact of characteristics of cigarette smoking on urinary tract cancer risk: a meta-analysis of epidemiologic studies. 89. 2000:630-9.

27. Malarcher A.M., Schulman J., Epstein L.A. *et al*. Methodological issues in estimating smoking-attributable mortality in the United States. AJE 2000; 152(6):573-84.

28. Calle E.E., Thun M.J., Petreli J.M., Rodriguez C., Heath C.W. Body-Mass Index and mortality in a prospective cohort of U.S. adults. NEJM 1999; 341:1097-105.

29. Hu F.B., Stampfer M.J., Manson J.E. *et al*. Trends in the incidence of coronary heart disease and changes in diet and lifestyle in women. NEJM 2000; 343:530-7.

30. Field A.E., Coakley E.H., Must A. *et al*. Impact of overweight on the risk of developing common chronic diseases during a 10-year period. Arch Intern Med 2001; 161:1581-6.

31. McPhillips J.B., Barrett-Connor E., Wingard D.L. Cardiovascular disease risk factors prior to the diagnosis of impaired glucose tolerance and non-insulin-dependent diabetes mellitus in a community of older adults. AJE 1990; 131(3):443-53.

32. Carey V.J., Walters E.E., Colditz G.A. *et al*. Body fat distribution and risk of non-insulin-dependent diabetes mellitus in women. The Nurses Health Study. AJE 1997; 145(7):614-9.

33. Njolstad I., Arnesen E., Lund-Larsen P.G. Smoking, serum lipids, blood pressure, and sex differences in myocardial infarction. A 12-year follow-up of the Finnmark Study. Circulation 1996; 93:450-6.

34. Kaye S.A., Folsom A.R., Sprafka J.M., Prineas R.J., Wallace R.B. Increased incidence of diabetes mellitus in relation to abdominal adiposity in older women. JCE 1991; 44(3):329-34.

35. Chan J.M., Rimm E.B., Colditz G.A., Stampfer M.J., Willett W.C. Obesity, fat distribution, and weight gain as risk factors for clinical diabetes in men. Diabetes Care 1994; 17(9):961-9.

36. Hart C.L., Hole D.J., Davey Smith G. Comparison of risk factors for stroke incidence and stroke mortality in 20 years of follow-up in men and women in the Renfrew/Paisley Study in Scotland. Stroke 2000; 31:1893-6.

37. Njolstad I., Arnesen E., Lund-Larsen P.G. Sex differences in risk factors for clinical diabetes mellitus in a general population: a 12-year follow-up of the Finnmark Study. AJE 1998; 147(1):49-58.

38. Rehm J.T., Bondy S.J., Sempos C.T., Vuong C.V. Alcohol consumption and coronary disease morbidity and mortality. AJE 1997; 146(6):495-501.

39. Bergstrom A., Pisani P., Tenet V., Wolk A., Adami H-O. Overweight as an avoidable cause of cancer in Europe. Int J Cancer 2001; 91:421-30.

40. Fiebach N.H., Hebert P.R., Stampfer M.J. *et al*. A prospective study of high blood pressure and cardiovascular disease in women. AJE 1989; 130(4):646-54.

41. MRFIT Research Group. Relationship between baseline risk factors and coronary heart disease and total mortality in the Multiple Risk Factor Intervention Trial. Prev Med 1986; 15:254-73.

42. Wilson P.W.F., Anderson K.M., Castelli W.P. Twelve-year incidence of coronary heart disease in middle-aged adults during the era of hypertensive therapy: the Framingham Offspring Study. Am J Med 1991; 90:11-6.

43. Colditz G.A., Bonita R., Stampfer M.J. *et al*. Cigarette smoking and risk of stroke in middle-aged women. NEJM 1988; 318(15):937-41.

44. Robbins A.S., Manson J.E., Lee I-M., Satterfield Z., Hennekens C.H. Cigarette smoking and stroke in a cohort of U.S. male physicians. Ann Intern Med 1994; 120:458-62.

45. Tverdal A., Thelle D., Stensvold I., Leren P., Bjartveit K. Mortality in relation to smoking history: 13 years'follow-up of 68,000 Norwegian men and women 35-49 years. J Clin Epid 1993; 46(5):475-87.

46. Slattery M, Samowtiz W, Ma K *et al*. CYP1A1, cigarette smoking, and colon and rectal cancer. AJE 2004; 160(9):842-52.

47. Viswanathan A, Feskanich D, De Vivo I *et al*. Smoking and the risk of endometrial cancer: Results from the Nurses' Health Study. Int J Cancer 2005; 114:996-1001.

48. Zhang B, Ferrence R, Cohen J *et al*. Smoking Cessation and Lung Cancer Mortality in a Cohort of Middle-aged Canadian Women. Ann Epidemiol 2005; 15(4):302-9.

49. Zeegers M, Tan F, Dorant E, van Den Brandt P. The impact of characteristics of cigarette smoking on urinary tract cancer risk: a meta-analysis of epidemiologic studies. Cancer 2000; 89(3):630-9.

50. Malarcher A.M., Schulman J., Epstein L.A. *et al*. Methodological issues in estimating

smoking-attributable mortality in the United States. Am J Epi 2000; 152(6):573-84.

51. Calle E.E., Thun M.J., Petreli J.M., Rodriguez C., Heath C.W. Body-Mass Index and mortality in a prospective cohort of U.S. adults. NEJM 1999; 341:1097-105.

52. Hu F.B., Stampfer M.J., Manson J.E. *et al*. Trends in the incidence of coronary heart disease and changes in diet and lifestyle in women. NEJM 2000; 343:530-7.

53. Field A.E., Coakley E.H., Must A. *et al*. Impact of overweight on the risk of developing common chronic diseases during a 10-year period. Arch Intern Med 2001; 161:1581-6.

54. McPhillips J.B., Barrett-Connor E., Wingard D.L. Cardiovascular disease risk factors prior to the diagnosis of impaired glucose tolerance and non-insulin-dependent diabetes mellitus in a community of older adults. Am J Epi 1990; 131(3):443-53.

55. Carey V.J., Walters E.E., Colditz G.A. *et al*. Body fat distribution and risk of non-insulin-dependent diabetes mellitus in women. The Nurses Health Study. Am J Epi 1997; 145(7):614-9.

56. Njolstad I., Arnesen E., Lund-Larsen P.G. Smoking, serum lipids, blood pressure, and sex differences in myocardial infarction. A 12-year follow-up of the Finnmark Study. Circulation 1996; 93:450-6.

57. Kaye S.A., Folsom A.R., Sprafka J.M., Prineas R.J., Wallace R.B. Increased incidence of diabetes mellitus in relation to abdominal adiposity in older women. JCE 1991; 44(3):329-34.

58. Chan J.M., Rimm E.B., Colditz G.A., Stampfer M.J., Willett W.C. Obesity, fat distribution, and weight gain as risk factors for clinical diabetes in men. Diabetes Care 1994; 17(9):961-9.

59. Hart C.L., Hole D.J., Davey Smith G. Comparison of risk factors for stroke incidence and stroke mortality in 20 years of follow-up in men and women in the Renfrew/Paisley Study in Scotland. Stroke 2000; 31:1893-6.

60. Njolstad I., Arnesen E., Lund-Larsen P.G. Sex differences in risk factors for clinical diabetes mellitus in a general population: a 12-year follow-up of the Finnmark Study. Am J Epi 1998; 147(1):49-58.

61. Rehm J.T., Bondy S.J., Sempos C.T., Vuong C.V. Alcohol consumption and coronary disease morbidity and mortality. AJE 1997; 146(6):495-501.

62. Bergstrom A., Pisani P., Tenet V., Wolk A., Adami H-O. Overweight as an avoidable cause of cancer in Europe. Int J Cancer 2001; 91:421-30.

63. Fiebach N.H., Hebert P.R., Stampfer M.J. *et al*. A prospective study of high blood pressure and cardiovascular disease in women. AJE 1989; 130(4):646-54.

64. MRFIT Research Group. Relationship between baseline risk factors and coronary heart disease and total mortality in the Multiple Risk Factor Intervention Trial. Prev Med 1986; 15:254-73.

65. Wilson P.W.F., Anderson K.M., Castelli W.P. Twelve-year incidence of coronary heart disease in middle-aged adults during the era of hypertensive therapy: the Framingham Offspring Study. Am J Med 1991; 90:11-6.

66. Slobbe LCJ, Kommer GJ, Smit JM, Groen J, Meerding WJ, Polder JJ. Costs of illness in the Netherlands 2003 . 2006; RIVM Report 270751010 .
