# Supplementary material for: Lifetime Medical Costs of Obesity: Prevention No Cure for Increasing Health Expenditure
Source: PLoS Med. 2008 Feb 5;5(2):e29. doi: 10.1371/journal.pmed.0050029 (PMC2225430; doi:10.1371/journal.pmed.0050029)
Supplement: Supplementary file 2 [file pmed.0050029.sd002.pdf]

Appendix C: Additional results base case scenario

Table 1 displays undiscounted lifetime health care cost for the three cohorts (each consisting of 500 men and 500 women aged 20 at baseline) specified by disease in the base case scenario.

Table 1: lifetime health care costs specified by disease expressed in € (undiscounted)

	obese cohort	'healthy living cohort'	smoking cohort
Acute myocardial infarct	1,867,520	1,179,035	1,478,884
Angina pectoris	7,148,414	4,869,843	5,844,816
Chronic heart failure	4,562,464	5,673,602	6,306,442
Stroke	11,984,902	13,262,546	10,765,913
COPD	5,043,264	1,157,241	1,000,621
diabetes	1,809,012	2,407,625	9,113,443
arthritis of the hip	2,594,210	4,224,126	6,403,136
arthritis of the knee	2,483,856	3,827,755	4,494,567
dorsopathies	3,132,445	4,157,900	4,362,861
cancer of lung	2,707,362	201,365	169,092
cancer of rectum	214,108	317,386	319,323
cancer of colon	571,066	886,766	1,046,993
cancer of stomach	230,651	282,872	217,245
cancer of oesophagus	661,330	222,840	175,091
cancer of breast	893,150	1,139,185	1,369,033
cancer of prostate	569,719	970,905	803,990
cancer of larnynx	125,089	15,360	12,578
cancer of uriny bladder	410,315	285,724	216,664
cancer of kidney	109,876	97,564	104,457
cancer of pancreas	197,372	187,717	150,130
cancer of oral cavity	751,236	162,807	134,578
cancer of endometrium	223,585	293,849	620,440
All other diseases	171,440,566	235,625,990	194,638,422
TOTAL	219,731,509	281,450,000	249,748,719

On the following pages, graphs are displayed with the health care costs for the three cohorts over time specified by disease (undiscounted) in the base case scenario.

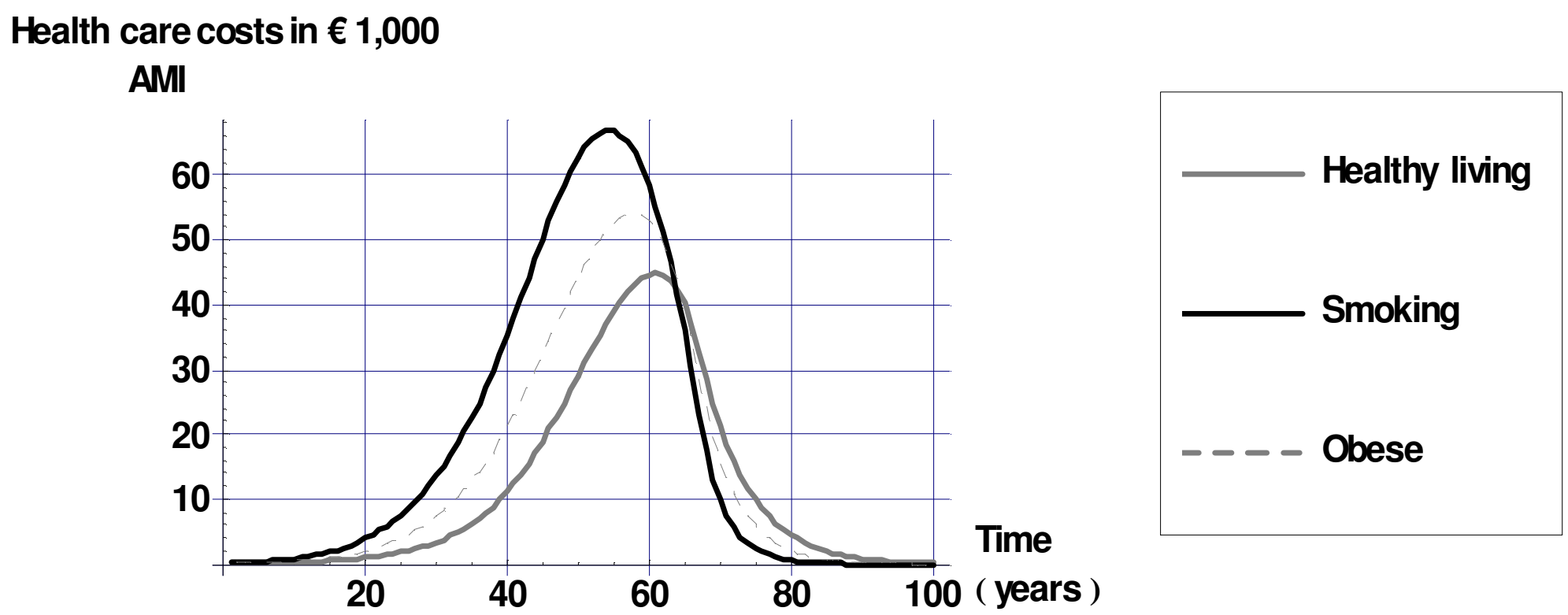

Figure 1: health care costs caused by acute myocardial infarct (AMI) for the three different cohorts over time

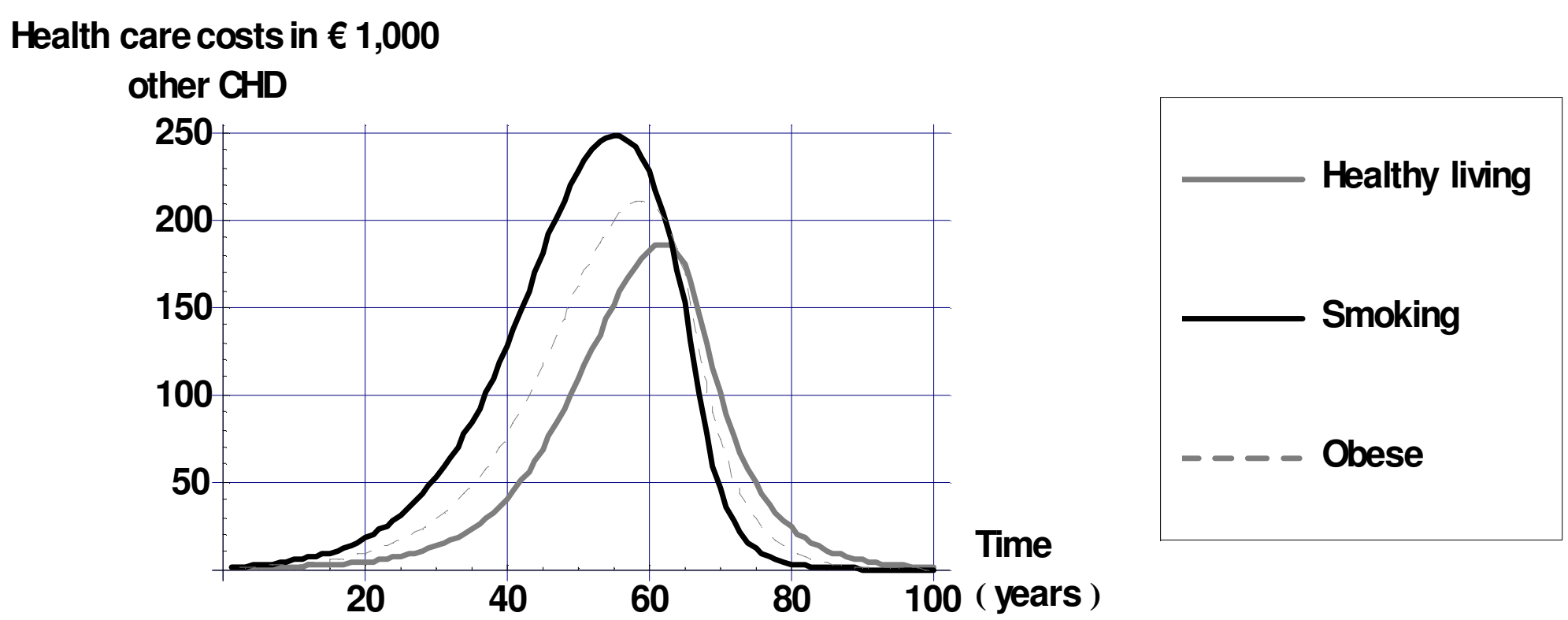

Figure 2: health care costs caused by angina pectoris (other CHD) for the three different cohorts over time

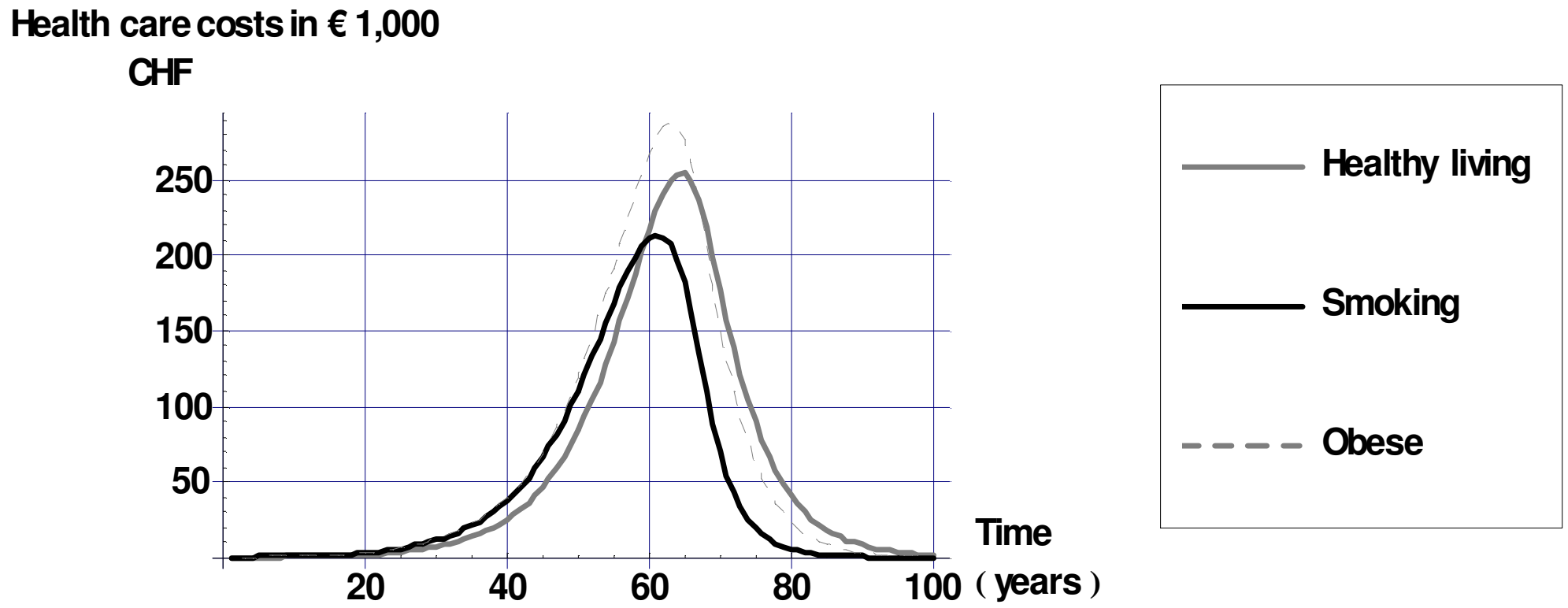

Figure 3: health care costs caused by chronic heart failure (CHF) for the three different cohorts over time

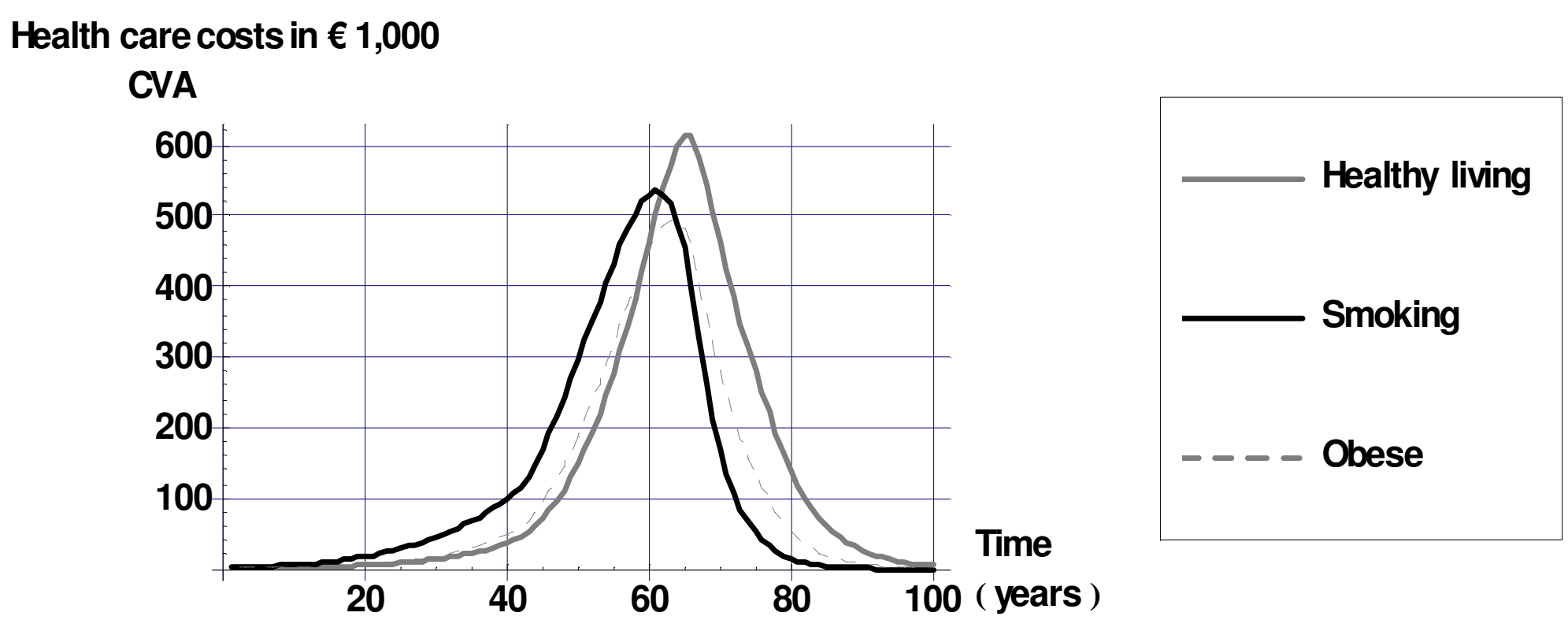

Figure 4: health care costs caused by stroke (CVA) for the three different cohorts over time

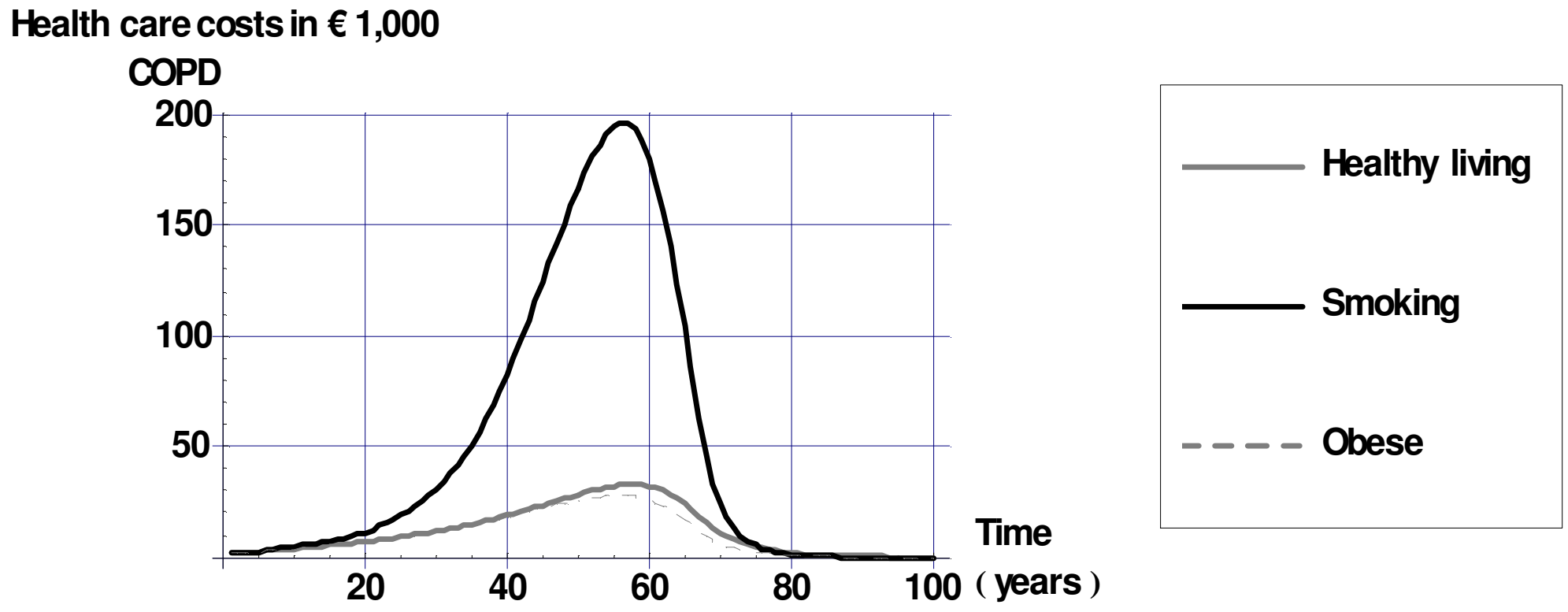

Figure 5: health care costs caused by COPD for the three different cohorts over time

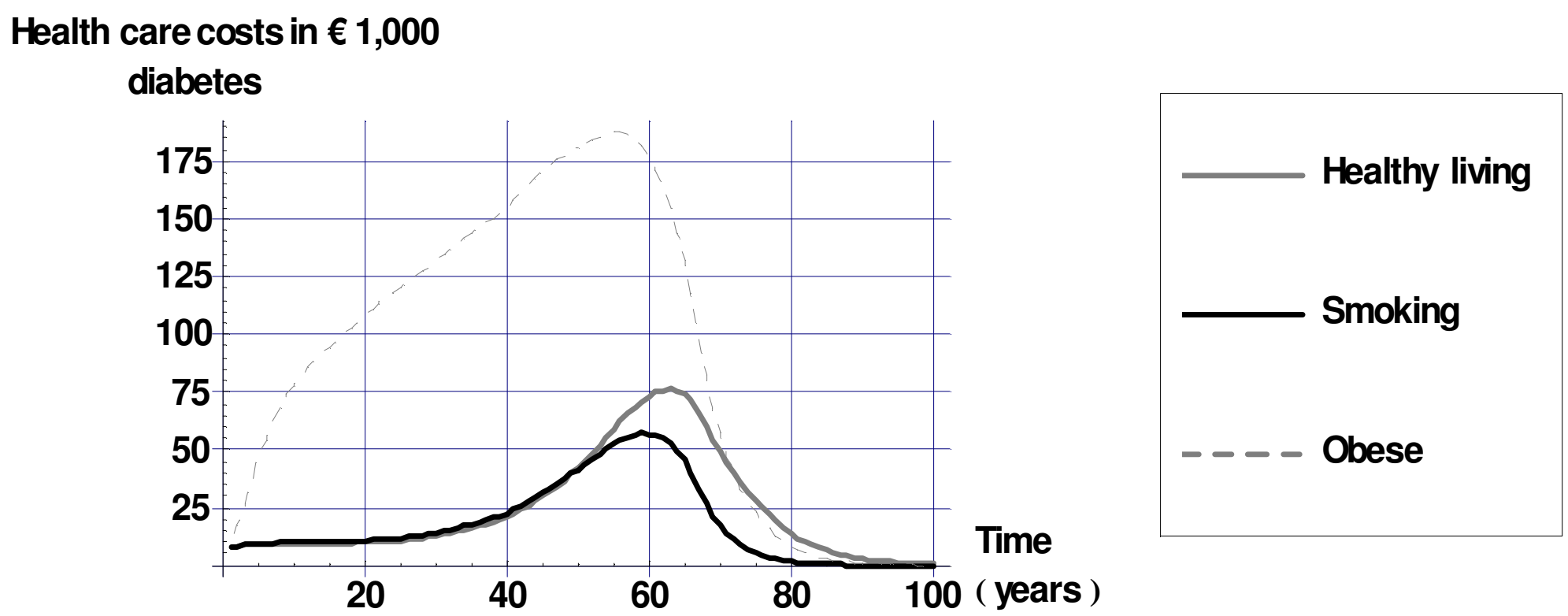

Figure 6: health care costs caused by diabetes for the three different cohorts over time

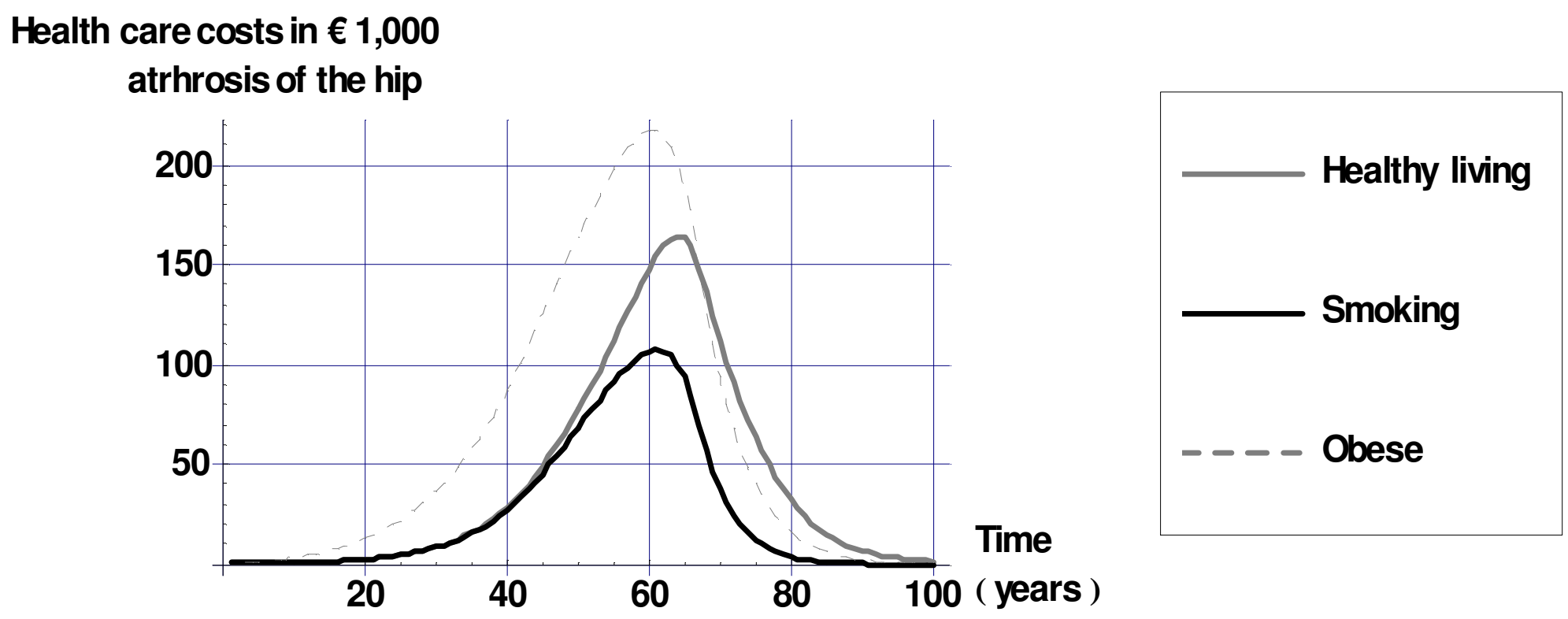

Figure 7: health care costs caused by arthritis of the hip for the three different cohorts over time

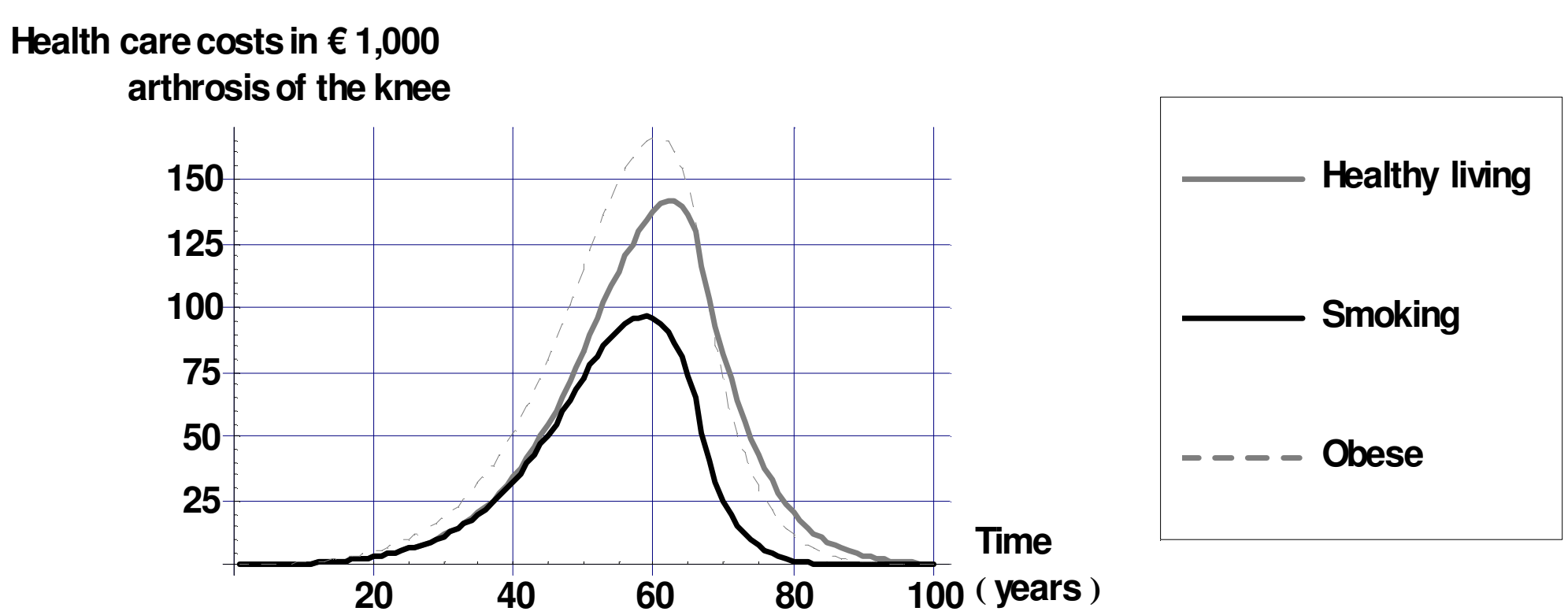

Figure 8: health care costs caused by arthritis of the knee for the three different cohorts over time

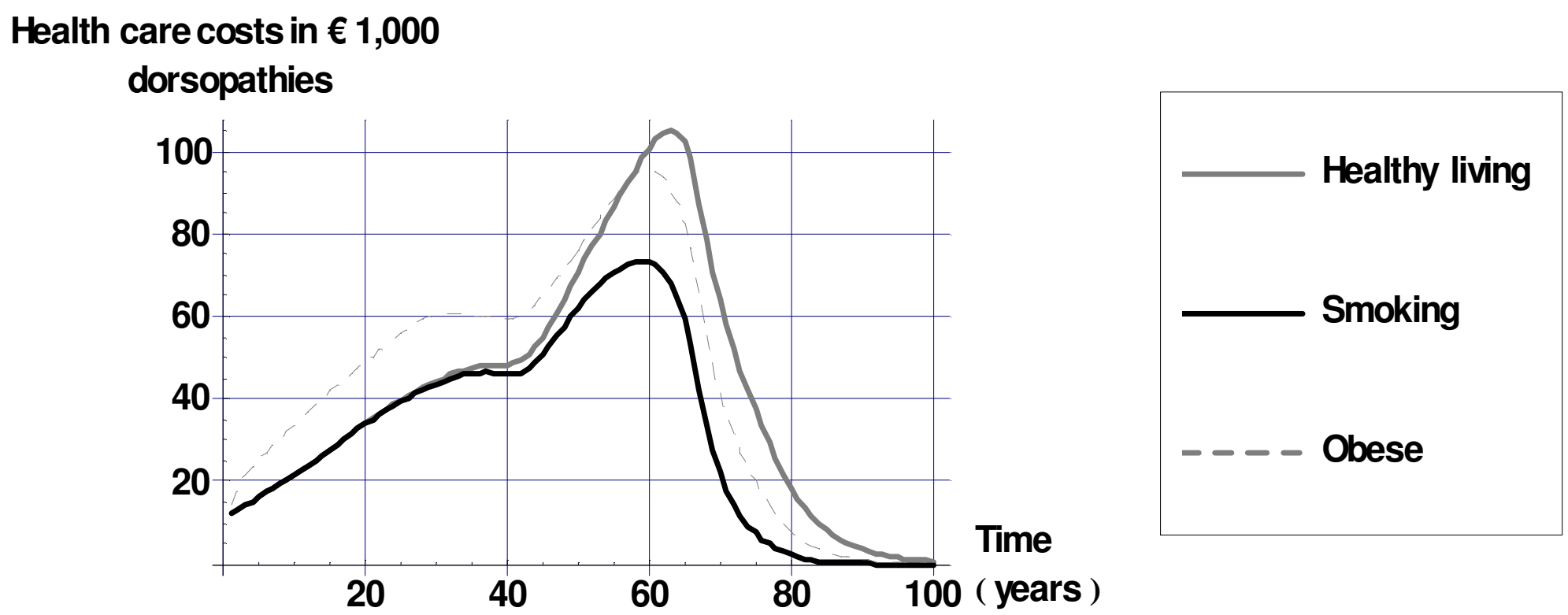

Figure 9: health care costs caused by low back pain (dorsopathies) for the three different cohorts over time

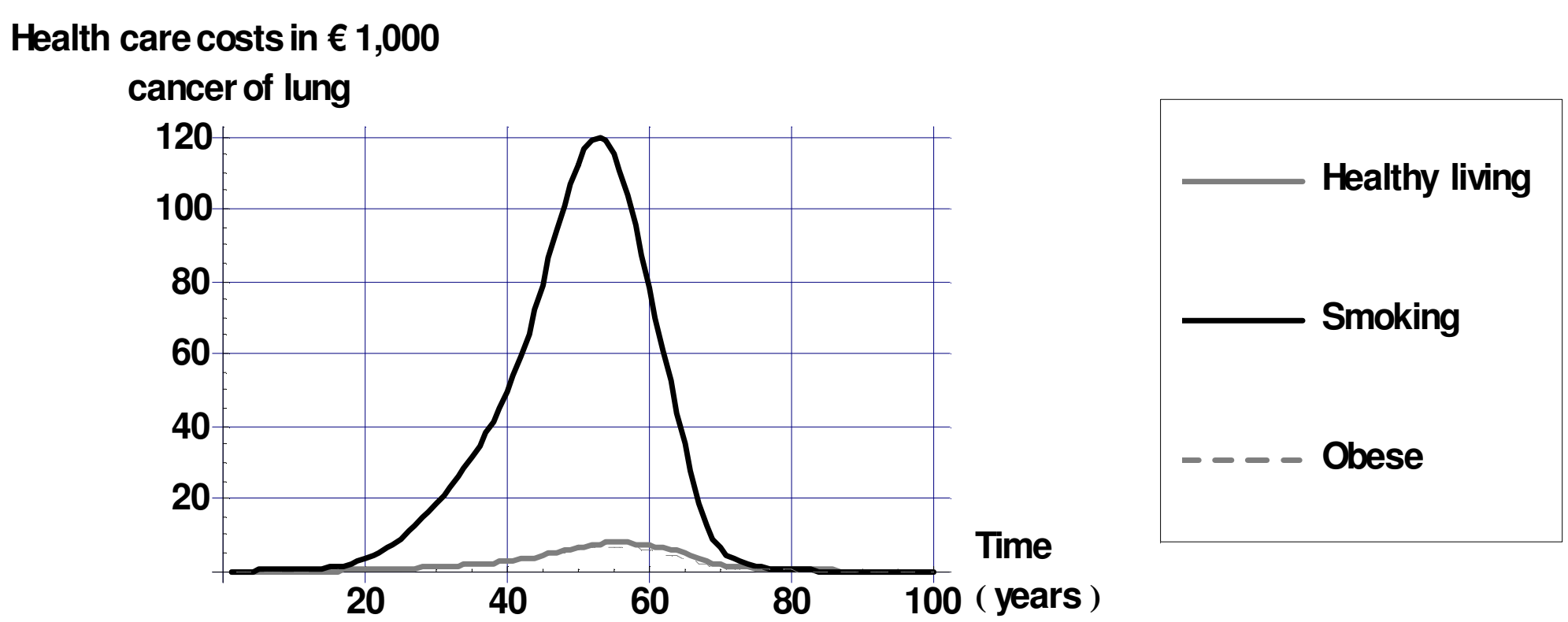

Figure 10: health care costs caused by lung cancer for the three different cohorts over time

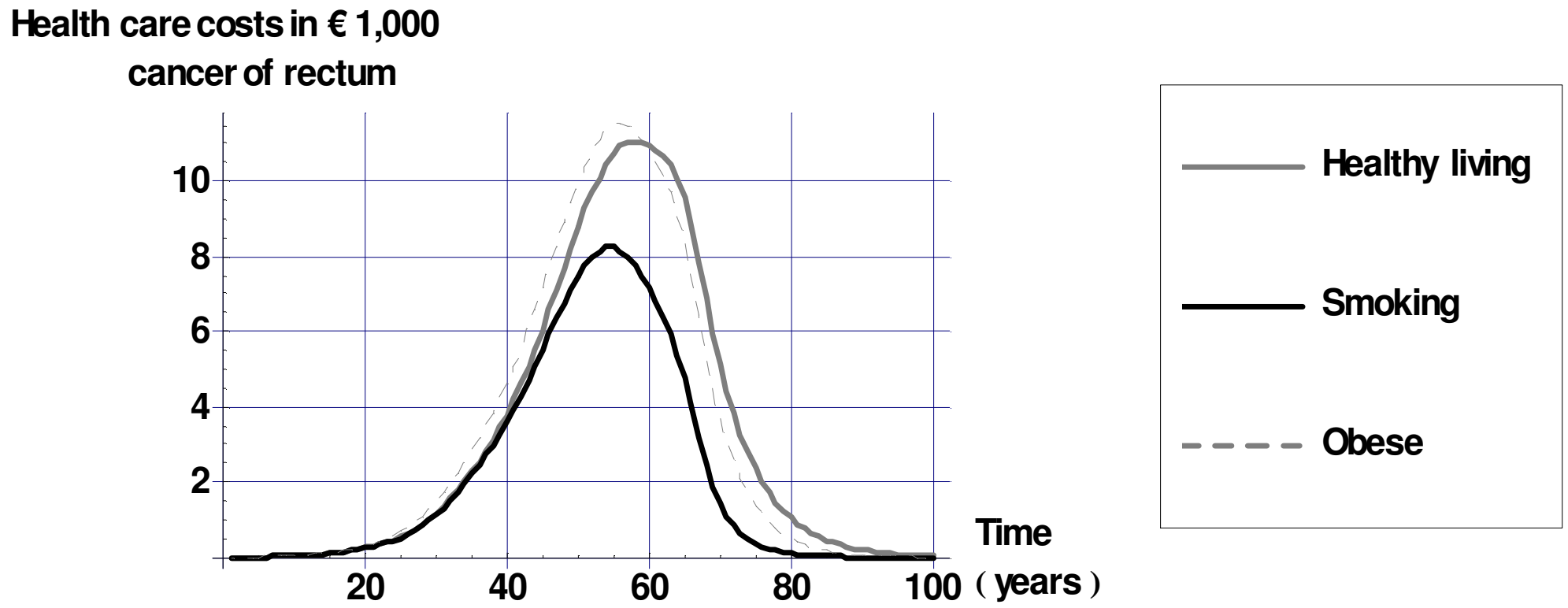

Figure 11: health care costs caused by rectum cancer for the three different cohorts over time

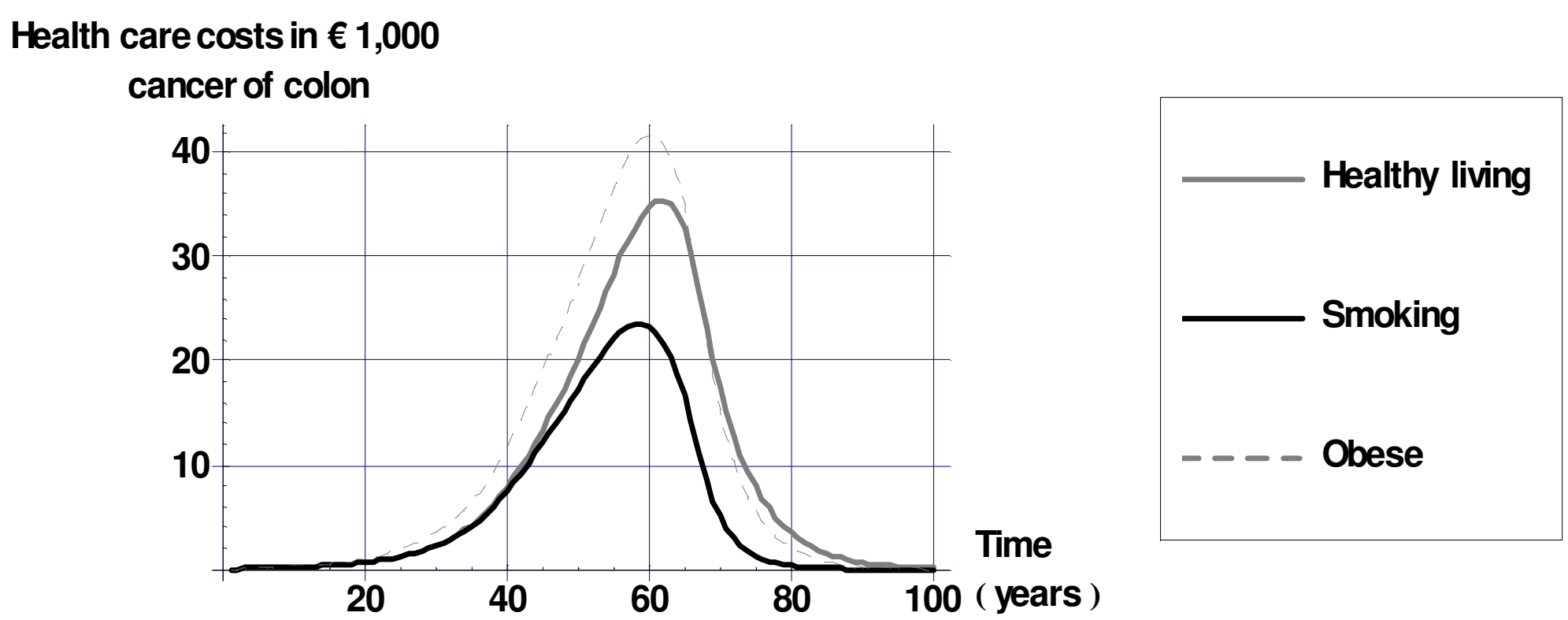

Figure 12: health care costs caused by colon cancer for the three different cohorts over time

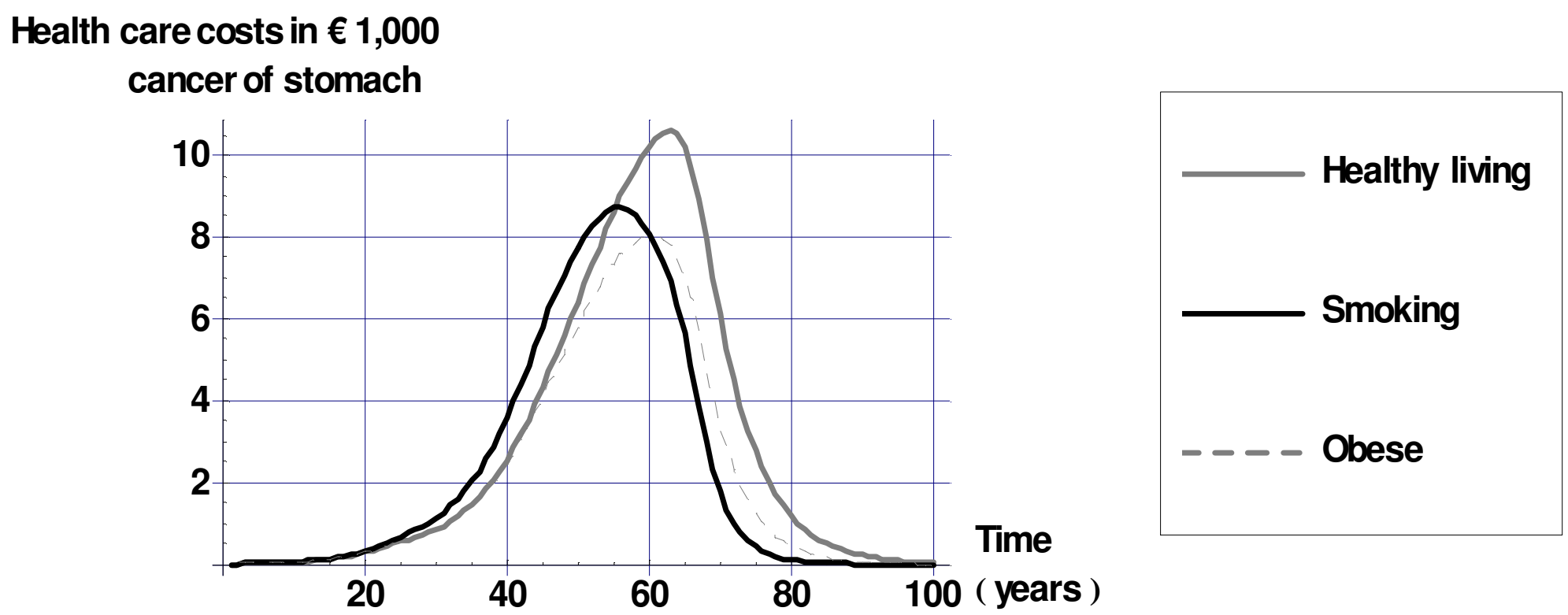

Figure 13: health care costs caused by stomach cancer for the three different cohorts over time

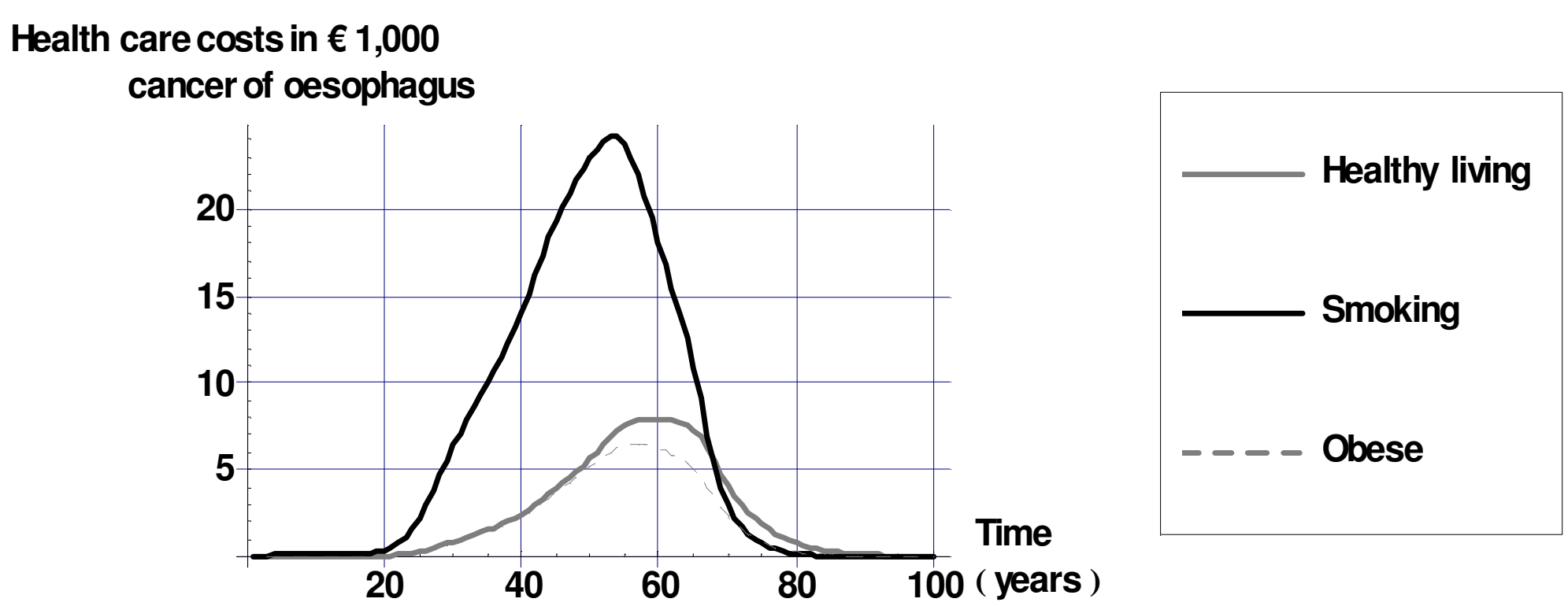

Figure 14: health care costs caused by oesophagus cancer for the three different cohorts over time

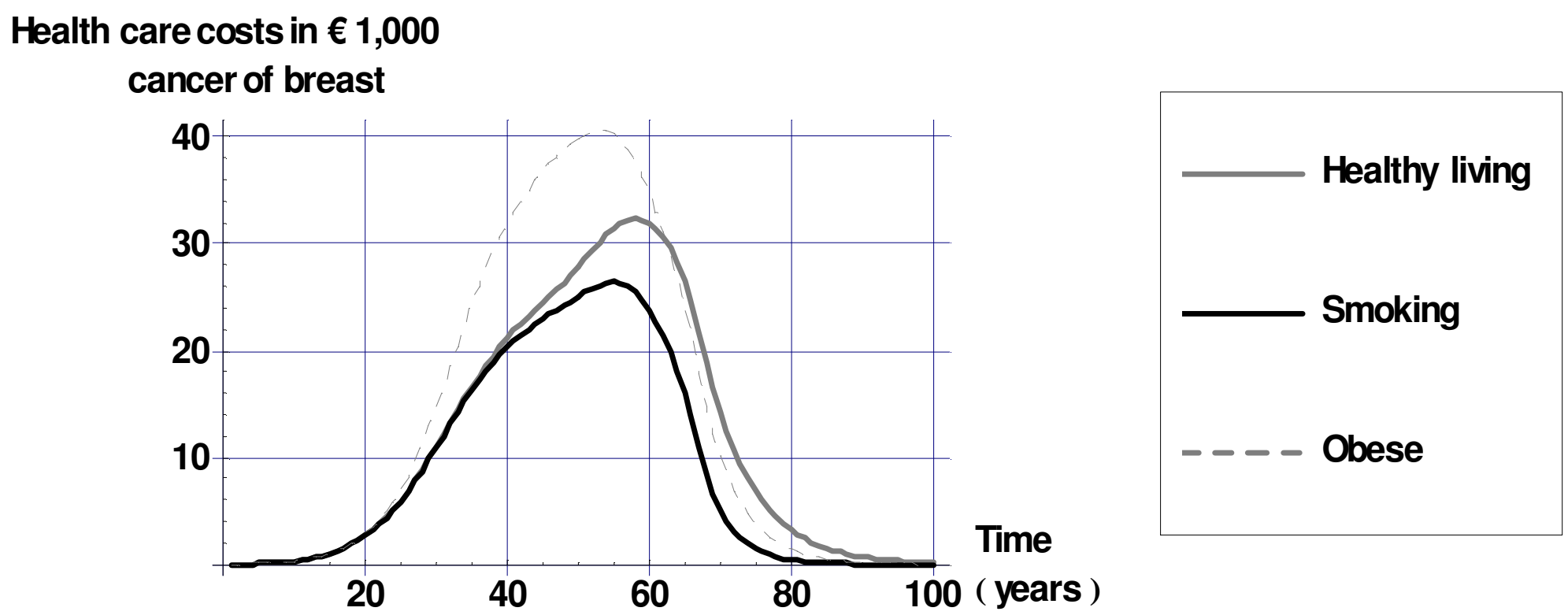

Figure 15: health care costs caused by breast cancer for the three different cohorts over time

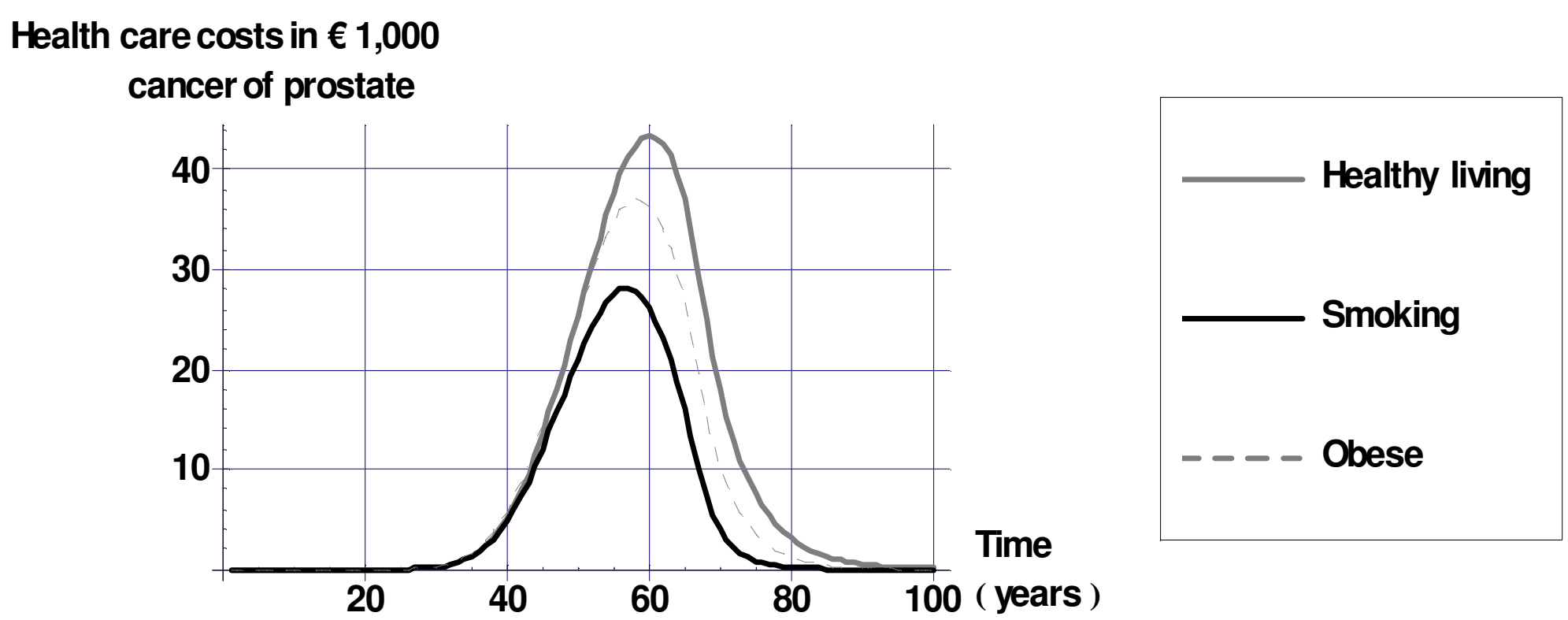

Figure 16: health care costs caused by prostate cancer for the three different cohorts over time

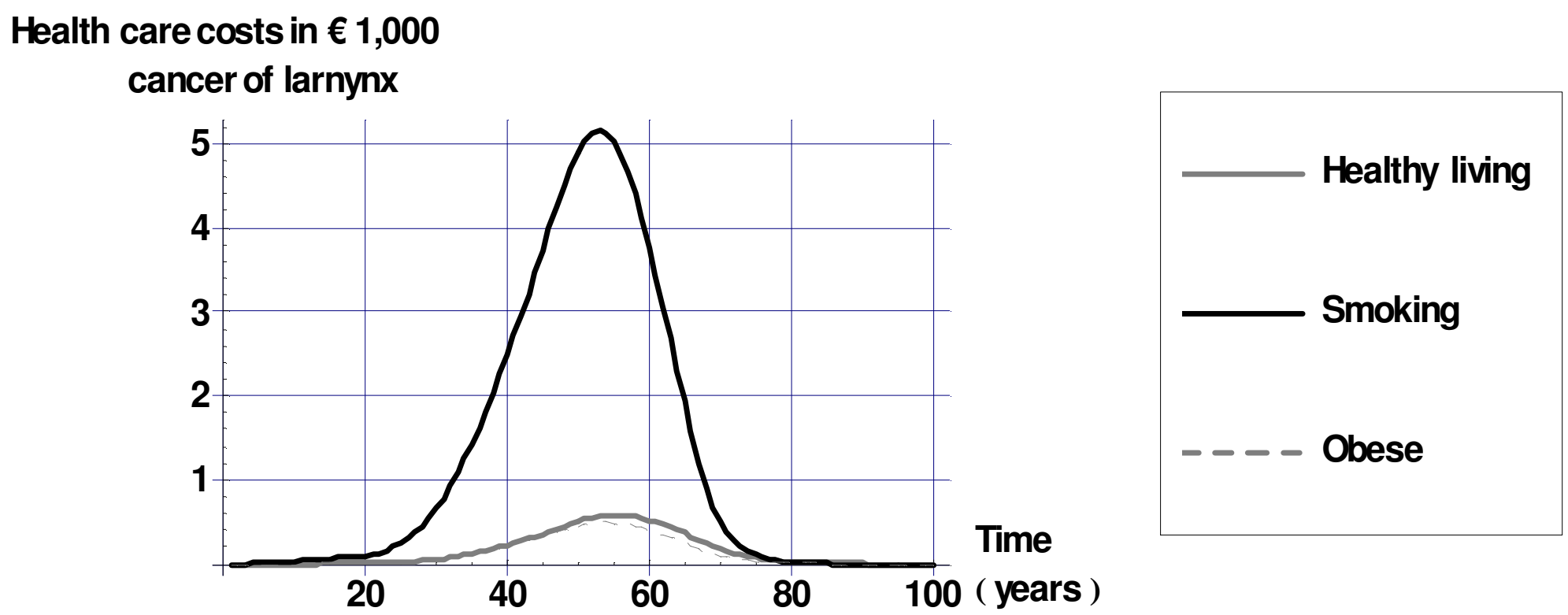

Figure 17: health care costs caused by larynx cancer for the three different cohorts over time

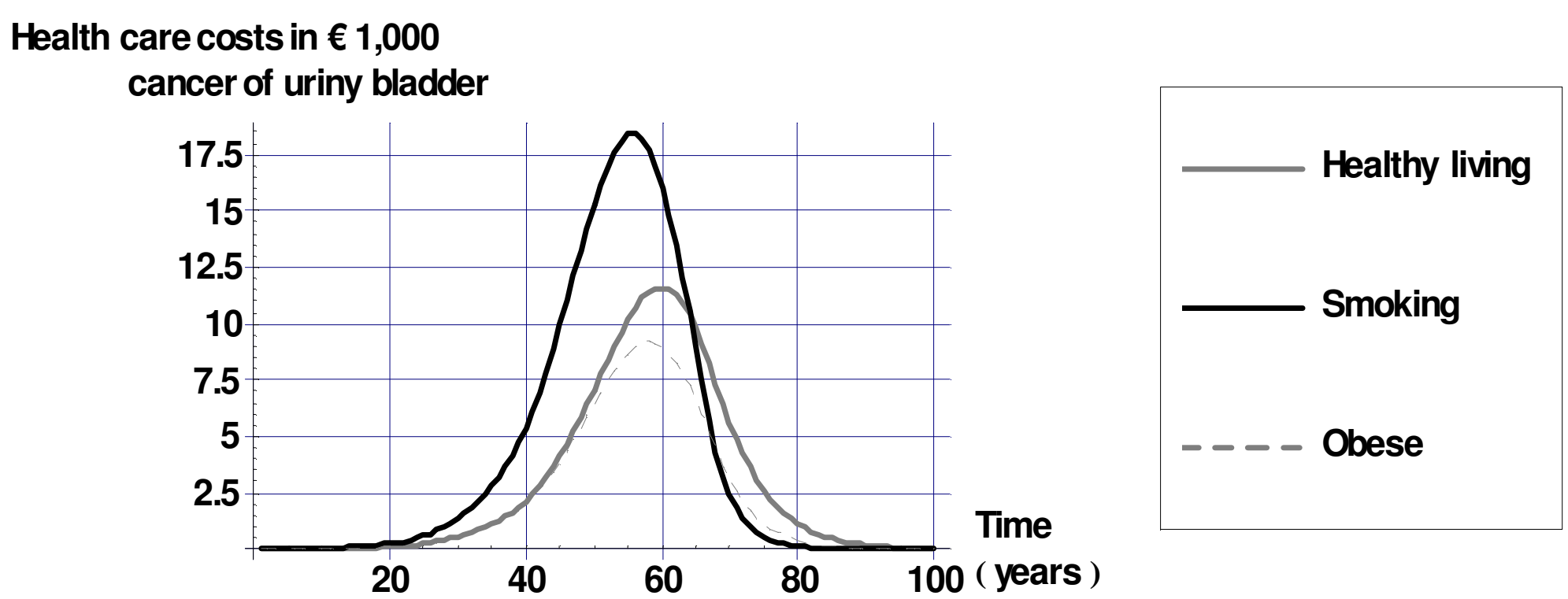

Figure 18: health care costs caused by uriny bladder cancer for the three different cohorts over time

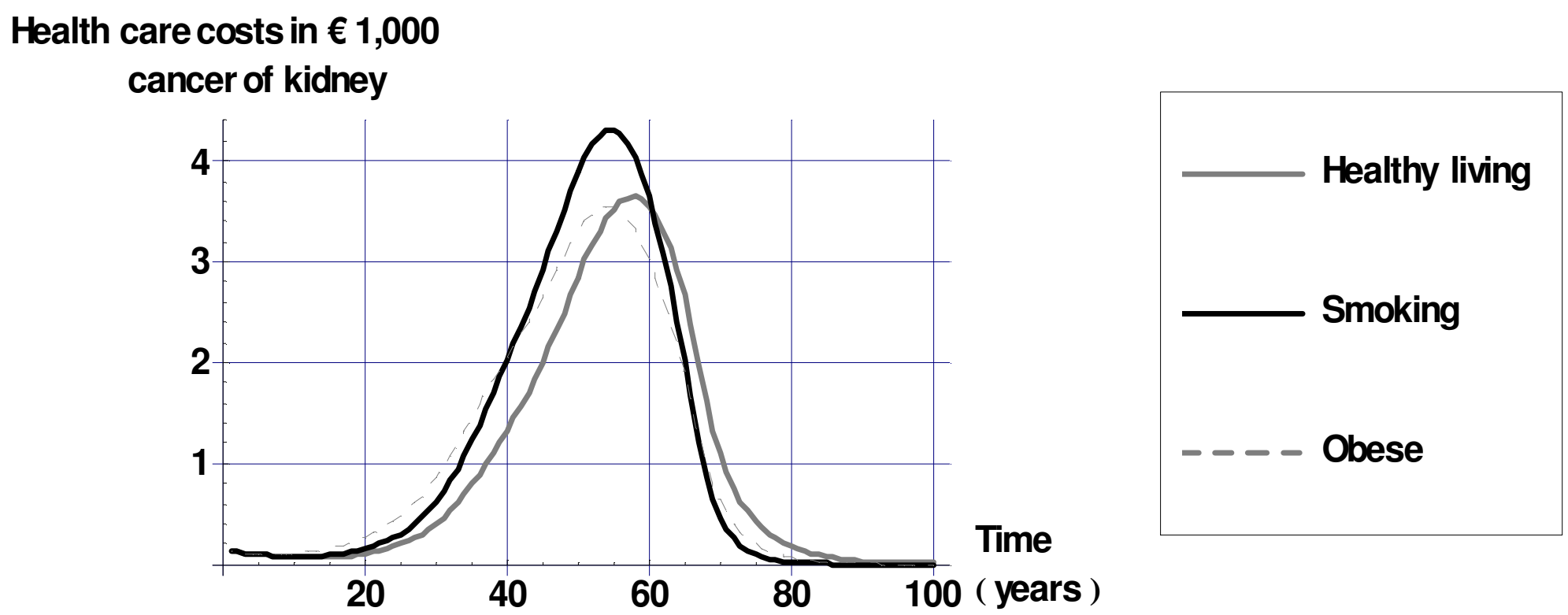

Figure 19: health care costs caused by kidney cancer for the three different cohorts over time

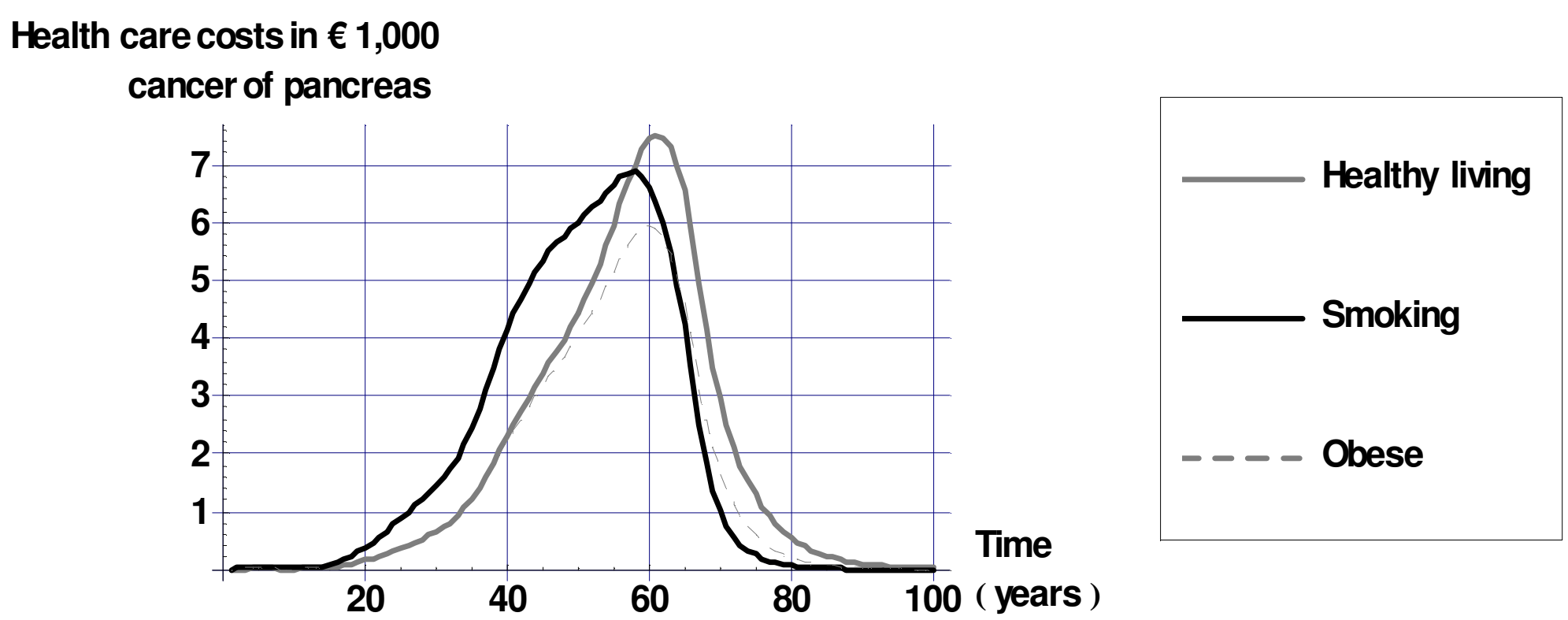

Figure 20: health care costs caused by pancreas cancer for the three different cohorts over time

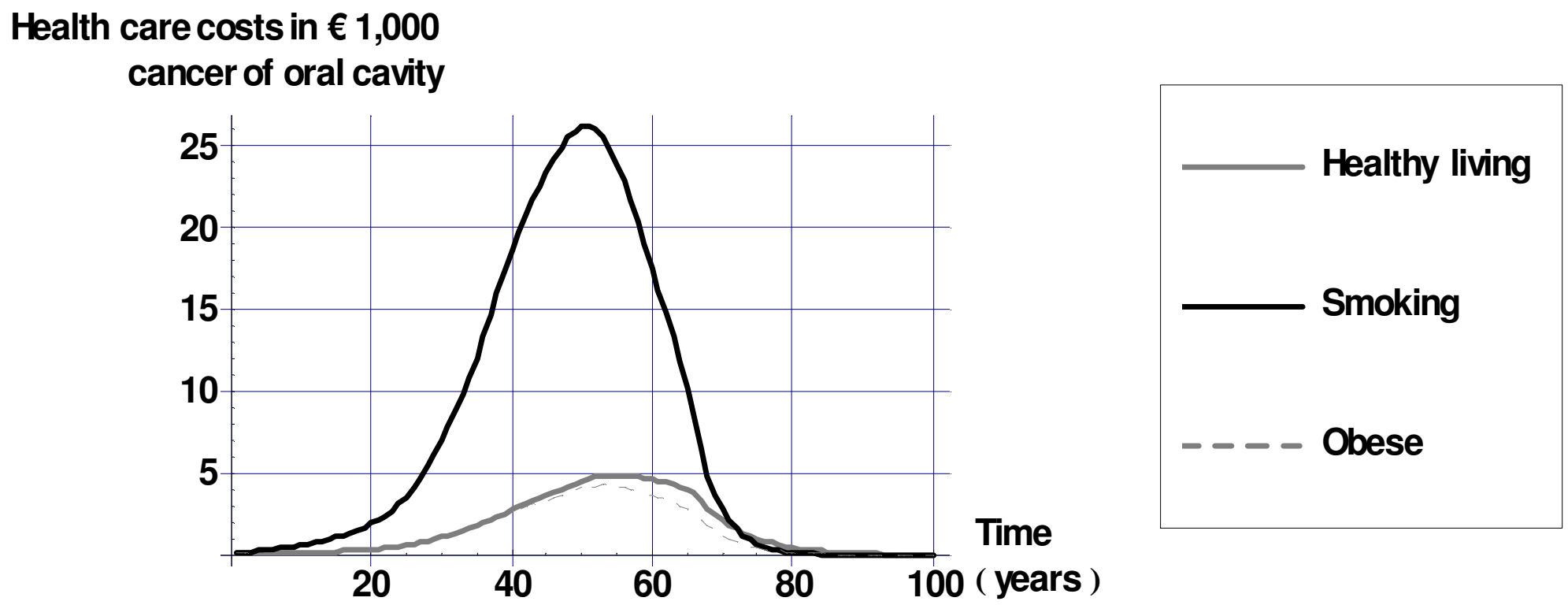

Figure 21: health care costs caused oral cavity cancer for the three different cohorts over time

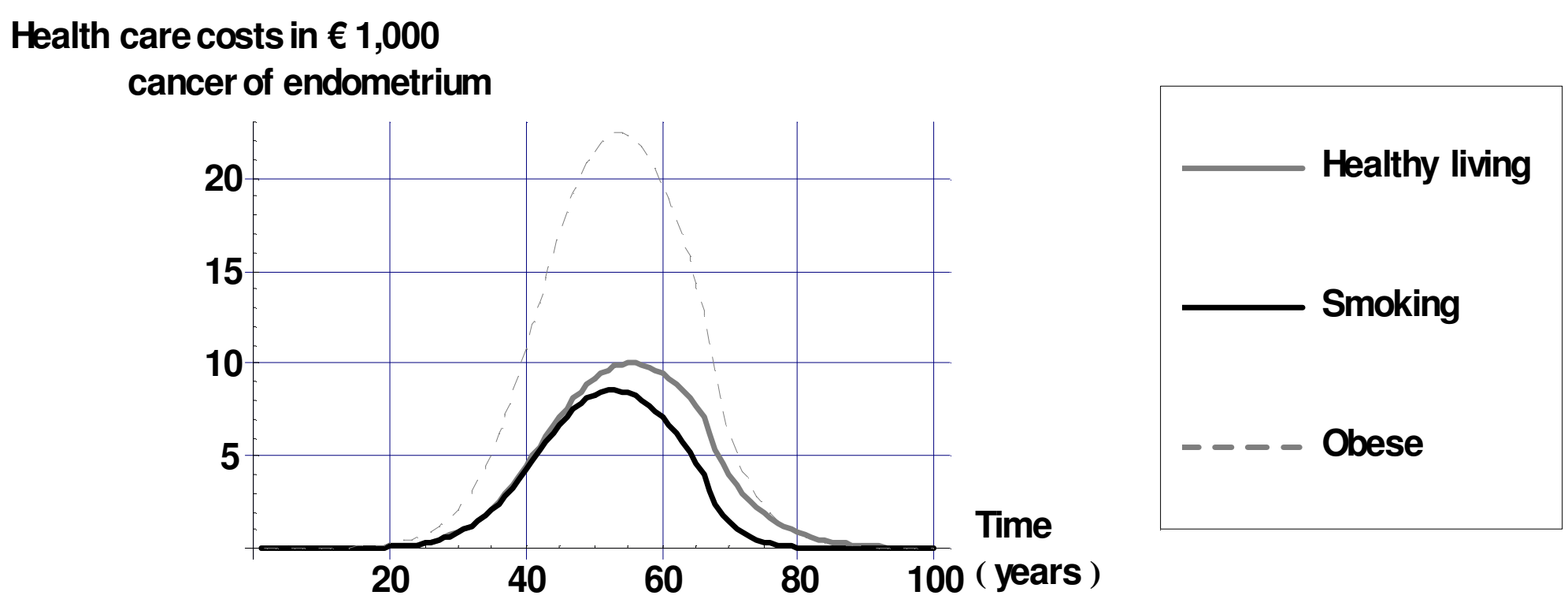

Figure 22: health care costs caused by endometrium cancer for the three different cohorts over time

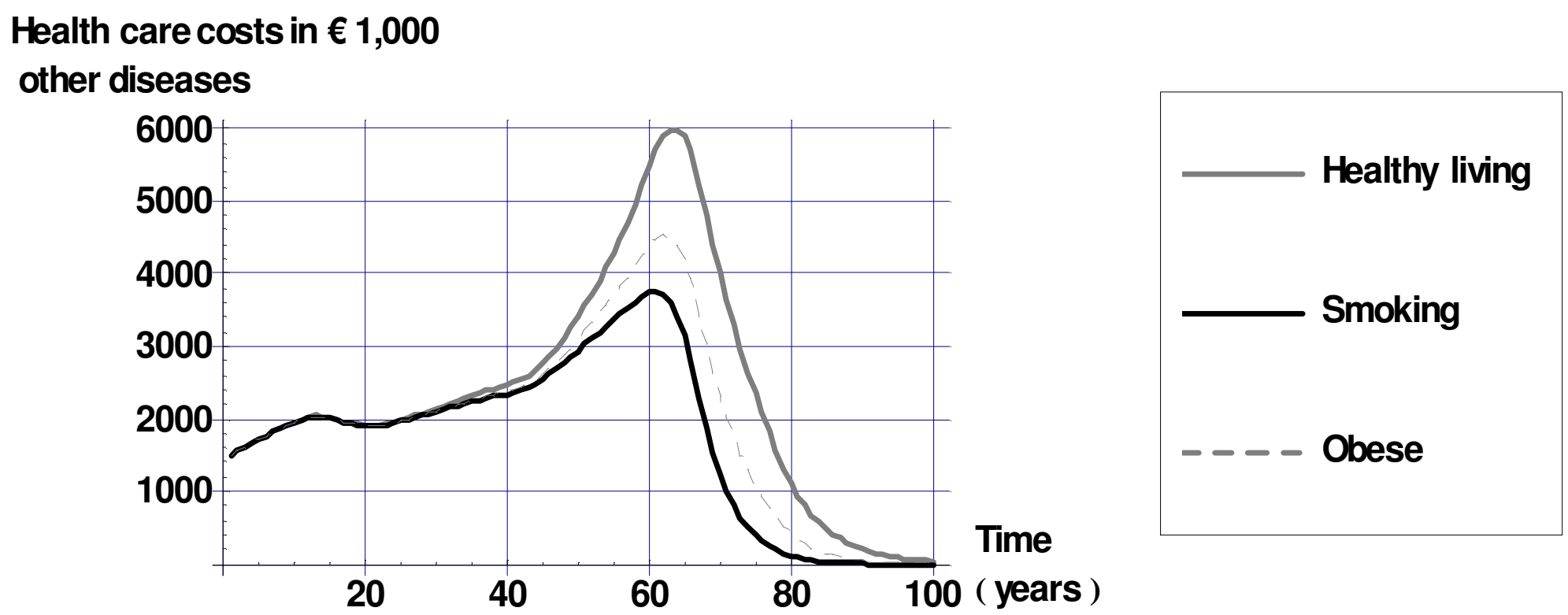

Figure 23: health care costs caused by all other diseases (not related to BMI and/or smoking) for the three different cohorts over time

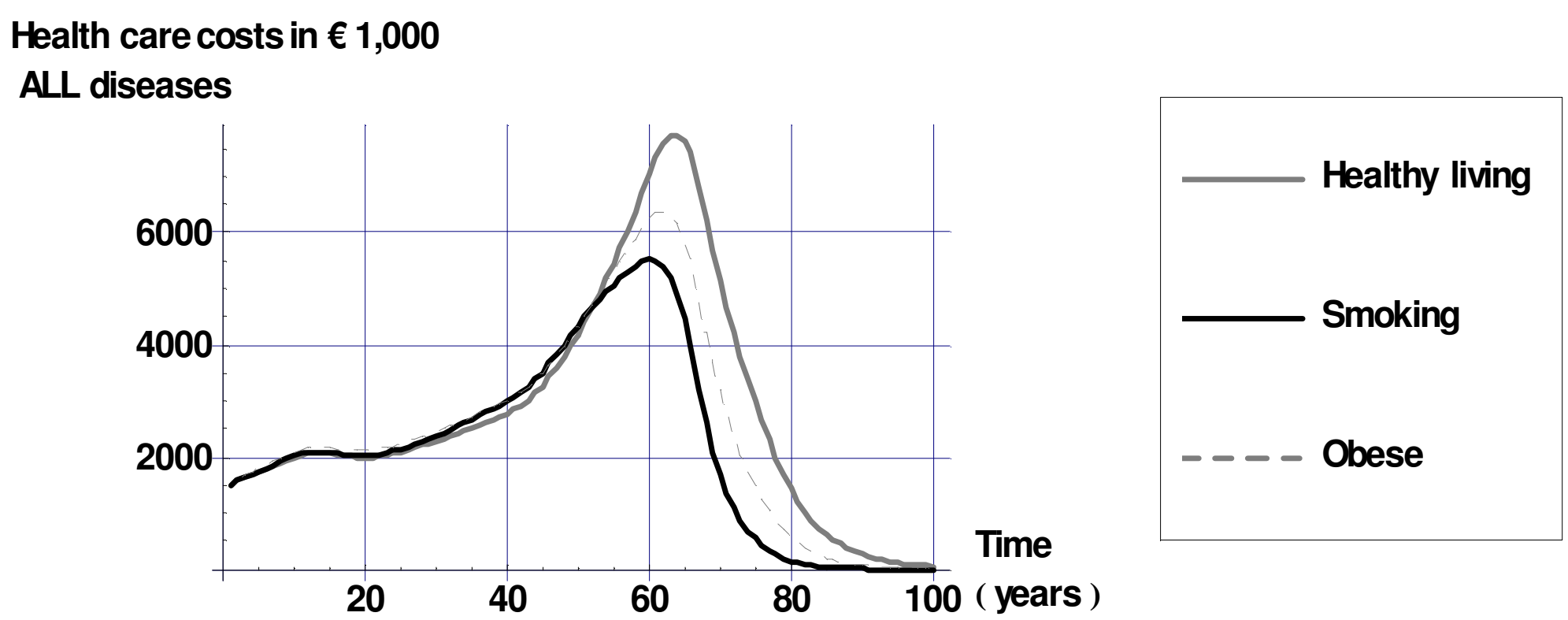

Figure 24: health care costs caused ALL diseases (both related and unrelated to BMI and/or smoking) for the three different cohorts over time
